# *Cryptococcus neoformans* Iron-Sulfur Protein Biogenesis Machinery Is a Novel Layer of Protection against Cu Stress

**DOI:** 10.1128/mBio.01742-17

**Published:** 2017-10-31

**Authors:** Sarela Garcia-Santamarina, Marta A. Uzarska, Richard A. Festa, Roland Lill, Dennis J. Thiele

**Affiliations:** a Duke University School of Medicine, Durham, North Carolina, USA; b Institut für Zytobiologie & Zytopathologie, Philipps-Universität, Marburg, Germany; c LOEWE Zentrum für Synthetische Mikrobiologie SynMikro, Marburg, Germany; d Department of Pharmacology and Cancer Biology, Department of Biochemistry, Duke University School of Medicine, Durham, North Carolina, USA; e Department of Molecular Genetics and Microbiology, Duke University School of Medicine, Durham, North Carolina, USA; University of Texas Health Science Center

**Keywords:** ABC transporters, copper toxicity, Cryptococcus neoformans, Fe-S cluster, copper ionophores, metalloproteins, mitochondria

## Abstract

Copper (Cu) ions serve as catalytic cofactors to drive key biochemical processes, and yet Cu levels that exceed cellular homeostatic control capacity are toxic. The underlying mechanisms for Cu toxicity are poorly understood. During pulmonary infection by the fungal pathogen *Cryptococcus neoformans*, host alveolar macrophages compartmentalize Cu to the phagosome, and the ability to detoxify Cu is critical for its survival and virulence. Here, we report that iron-sulfur (Fe-S) clusters are critical targets of Cu toxicity in both *Saccharomyces cerevisiae* and *C. neoformans* in a manner that depends on the accessibility of Cu to the Fe-S cofactor. To respond to this Cu-dependent Fe-S stress, *C. neoformans* induces the transcription of mitochondrial ABC transporter Atm1, which functions in cytosolic-nuclear Fe-S protein biogenesis in response to Cu and in a manner dependent on the Cu metalloregulatory transcription factor Cuf1. As Atm1 functions in exporting an Fe-S precursor from the mitochondrial matrix to the cytosol, *C. neoformans* cells depleted for Atm1 are sensitive to Cu even while the Cu-detoxifying metallothionein proteins are highly expressed. We provide evidence for a previously unrecognized microbial defense mechanism to deal with Cu toxicity, and we highlight the importance for *C. neoformans* of having several distinct mechanisms for coping with Cu toxicity which together could contribute to the success of this microbe as an opportunistic human fungal pathogen.

## IMPORTANCE

*C. neoformans* is an opportunistic pathogen that causes lethal meningitis in over 650,000 people annually. The severity of *C. neoformans* infections is further compounded by the use of toxic or poorly effective systemic antifungal agents as well as by the difficulty of diagnosis. Cu is a natural potent antimicrobial agent that is compartmentalized within the macrophage phagosome and used by innate immune cells to neutralize microbial pathogens. While the Cu detoxification machinery of *C. neoformans* is essential for virulence, little is known about the mechanisms by which Cu kills fungi. Here we report that Fe-S cluster-containing proteins, including members of the Fe-S protein biogenesis machinery itself, are critical targets of Cu toxicity and therefore that this biosynthetic process provides an important layer of defense against high Cu levels. Given the role of Cu ionophores as antimicrobials, understanding how Cu is toxic to microorganisms could lead to the development of effective, broad-spectrum antimicrobials. Moreover, understanding Cu toxicity could provide additional insights into the pathophysiology of human diseases of Cu overload such as Wilson’s disease.

## INTRODUCTION

*Cryptococcus neoformans* is a fungal pathogen that lives in the soil and on plants in the environment and which infects humans through inhalation. The lungs are the primary site of infection, where alveolar macrophages compartmentalize *C. neoformans* into the phagosome, a hostile environment that serves to dampen fungal growth and survival. *C. neoformans* cells that survive phagocytosis can escape macrophages through lytic or nonlytic exocytosis and can cause cryptococcal pneumonia or can enter the bloodstream and cross the blood-brain barrier, causing lethal meningitis (1–3). Each year, approximately 1 million cryptococcal infections are diagnosed, resulting in over 600,000 deaths per year, predominantly among immunocompromised individuals (4). Similarly to other opportunistic pathogens, *C. neoformans* must quickly adapt its life cycle to the constantly changing and challenging host microenvironments. Thus, the generation of a polysaccharide capsule, elaboration of enzymes such as melanin and urease, and the ability to adapt to excess or limited levels of factors required for growth are traits that influence the colonization, survival, and virulence of this organism within the host (5–8).

An important virulence trait that allows successful cryptococcosis is the ability of *C. neoformans* to sense and adapt to different host-driven copper (Cu) environments (9, 10). Cu is an essential transition metal required in many key biochemical processes, many of which are directly linked to virulence in *C. neoformans* (11). Thus, Cu is a cofactor for key enzymes involved in melanin formation, high-affinity Fe uptake, reactive oxygen species detoxification, and respiration (12–16). However, when present at high concentrations, Cu is also toxic. In this regard, *C. neoformans* Cu metalloregulatory transcription factor Cuf1 has been shown to be required for virulence, as it regulates the expression of genes required for both the acquisition and detoxification of Cu (17, 18). Under conditions of low Cu concentrations, Cuf1 activates the expression of genes encoding the *C*tr1 and Ctr4 Cu^+^ transporters and cell surface metalloreductases which, together, drive Cu acquisition. In response to high environmental Cu concentrations, Cuf1 activates the expression of genes encoding the Cu-buffering metallothioneins Mt1 and Mt2 (17). Cuf1, Mt1/Mt2, and Ctr1/4 are all virulence factors in mouse models of cryptococcosis, and their requirement for virulence depends on the infectious niche (9, 10). In the brain, Ctr1 and Ctr4 are critical for Cu acquisition and cell survival (10). Within alveolar macrophages, however, *C. neoformans* Mt1/2 expression is critical for survival of the elevated Cu levels encountered in the phagosomal compartment (9).

It has recently been recognized that Cu is used by host innate immune cells as a potent microbicide. Using X-ray microprobe analysis, it was demonstrated that Cu concentrations increase in the phagosome of peritoneal macrophages infected with *Mycobacterium* spp. (19). Furthermore, activated macrophages coordinately increase expression of the Ctr1 Cu^+^ importer at the plasma membrane and localize the ATPase ATP7A at the phagolysosomal membrane, resulting in the accumulation and compartmentalization of Cu in the phagosome (20). Defects in Cu^+^ exporters or in metallothionein expression sensitize both bacterial and fungal pathogens to macrophage killing mechanisms that are dependent on the toxicity of Cu (20–23). However, the precise mechanisms whereby Cu is toxic to organisms remain incompletely understood. Classically, due to its redox properties, Cu-associated toxicity was attributed to an increase in the intracellular levels of reactive oxygen species, which irreversibly damage DNA, lipids, and/or proteins. More recently, Cu toxicity mechanisms were revisited and it has been suggested that iron-sulfur (Fe-S) cluster interference is a significant mechanism for Cu toxicity in bacterial cells (24, 25). Hence, recent *in vivo* and *in vitro* studies in *Escherichia coli* demonstrated that Cu targets solvent-exposed Fe-S clusters of dehydratases involved in the synthesis of branched-chain amino acids and the *E. coli* Fe-S cluster biogenesis protein IscA (24, 26). Additional work performed *in vitro* demonstrated that Cu^+^ destabilizes Fe-S clusters from the bacterial SufU protein (27), the major scaffold used by the sulfur assimilation (SUF) system for Fe-S cluster assembly and transfer to target proteins, and from mammalian ISCA1/2 and GLRX5, mitochondrial proteins involved in Fe-S cluster trafficking (28).

Here we report that Fe-S cluster-containing proteins and the Fe-S protein assembly machinery itself are critical targets for Cu toxicity in *C. neoformans* and in *Saccharomyces cerevisiae*. In *S. cerevisiae*, Cu targets Fe-S clusters, including several components of the cytosolic Fe-S protein assembly (CIA) machinery, with sensitivity that depends on the solvent accessibility of the cofactor to Cu. Unexpectedly, *C. neoformans* responded to elevated Cu levels by activating the expression of the *ATM1* gene, encoding a mitochondrial ABC transporter that mobilizes a precursor for Fe-S biogenesis to the cytosol (29–31), in a Cuf1-dependent manner. Atm1 depletion resulted in a growth defect under conditions of Cu stress, even in the presence of the Cu-buffering metallothioneins. These results highlight the importance for *C. neoformans* of implementing several mechanisms for coping with Cu toxicity, which together could contribute to the survival of this pathogen in the presence of the host innate immune system.

## RESULTS

### Atm1, a predicted ABC transporter with functions in the ISC export machinery, is regulated by Cuf1 during Cu stress in *C. neoformans.*

**FIG 1:**
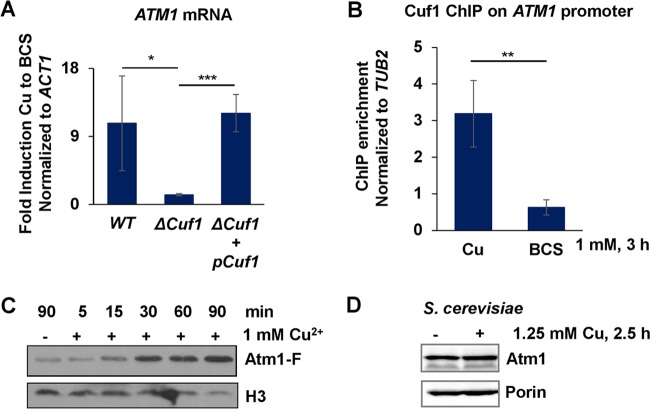
Expression of the mitochondrial ABC transporter Atm1 is induced by Cu in a Cuf1-dependent manner in *C. neoformans*. (A) Exponentially growing cultures of *C. neoformans* strains H99 (DTY758), Δ*Cuf1* (DTY761), and Δ*Cuf1* with complementing Cuf1 (DTY762) were treated with 1 mM BCS and 1 mM Cu for 3 h. Gene expression was analyzed by qRT-PCR with specific primers for *ATM1* and *ACT1* (used for data normalization). *n* = 4 (one-way repeated-measures ANOVA test, *P* = 0.02). (B) Exponentially growing cultures of *C. neoformans* strains Cuf1-FLAG (DTY762) and H99 (DTY758) were treated with 1 mM CuSO_4_ or 1 mM BCS. ChIP was performed using anti-FLAG antibody, and Cuf1 occupancy was analyzed by qRT-PCR with primers for the promoter regions of the *ATM1* and *TUB2* genes. *C. neoformans* H99 was used as a negative control. *n* = 3 (Student’s *t* test of paired samples, *P* = 0.002). (C) Cellular protein extracts were obtained from exponentially growing cultures of *C. neoformans* strain Atm1-F (DTY947) that had been left untreated (unt) or had been treated with 1 mM Cu during the indicated times and analyzed by SDS-PAGE and immunoblotting with antibodies against FLAG and histone 3 (H3; loading control). (D) *S. cerevisiae* Atm1 protein expression is not affected by Cu stress. Cellular protein extracts were obtained from untreated or Cu (1.25 mM)-treated exponentially growing cultures of *S. cerevisiae* strain W303A (wild type [WT]) and analyzed by SDS-PAGE and immunoblotting with antibodies against Atm1 and porin (loading control).

Given the importance of the Cu detoxification machinery in *C. neoformans* pulmonary infection (9), we characterized the transcriptome of *C. neoformans* in response to elevated Cu concentrations to identify additional pathways relevant for handling toxic Cu concentrations. One gene activated during Cu stress corresponds to *CNAG_04358*, which codes for a homologue of the previously characterized *S. cerevisiae* mitochondrial inner membrane ATP-binding cassette (ABC) transporter Atm1. In *S. cerevisiae*, this protein exports from the mitochondrial matrix to the cytosol an unknown, sulfur-containing component which is required for maintaining cellular Fe homeostasis and is utilized by the CIA machinery for maturation of cytosolic and nuclear Fe-S proteins (29, 30, 32). In *C. neoformans*, expression of genes in response to Cu limitation as well as Cu excess is dependent on metalloregulatory transcription factor Cuf1 (17). To ascertain whether *C. neoformans ATM1* expression during Cu stress is Cuf1 dependent, *ATM1* mRNA levels were analyzed and quantitative real-time reverse transcription-PCR (qRT-PCR) analysis of the wild-type strain, mutant *ΔCuf1*, and a *ΔCuf1* strain complemented with wild-type Cuf1 was performed (Fig. 1A). *ATM1* mRNA levels were significantly reduced in the *ΔCuf1* strain compared with the parental wild-type and complemented *ΔCuf1* strains. To further address whether *C. neoformans ATM1* belongs to the Cuf1 regulon, Cuf1 occupancy on the *ATM1* promoter was analyzed by chromatin immunoprecipitation-PCR (ChIP-PCR) in wild-type cells that were treated either with Cu or with the Cu chelator bathocuproine disulfonic acid (BCS) (Fig. 1B). The results showed that Cuf1 was significantly enriched at the *ATM1* promoter during Cu exposure compared to the results seen under conditions of Cu limitation. To validate that increased *ATM1* mRNA levels result in increased Atm1 protein levels, the Atm1 protein was fused with a carboxyl-terminal 4× FLAG epitope tag (Atm1-F) under the control of the native *ATM1* promoter to generate a functional fusion protein (see Fig. S1A in the supplemental material). After treatment with 1 mM Cu, Atm1-F levels, analyzed by immunoblotting, increased as early as 15 min after Cu stress and continued to accumulate for at least 90 min (Fig. 1C). Together, these results demonstrate that Cu enhances *C. neoformans* Cuf1-dependent *ATM1* expression both at the mRNA level and at the protein level.

10.1128/mBio.01742-17.2 FIG S1  The *C. neoformans* Atm1 protein sequence is highly conserved. (A) Atm1-F is a functional fusion protein. Strains H99 (DTY758), *ΔCuf1* (DTY761), and Atm1-F (DTY947) were spotted on SC plates with or without the indicated amount of Cu. (B) Multisequence alignment of Atm1 homologues was created by the use of Multalin software (F. Corpet, Nucleic Acids Res 16:10881–10890, 1988, https://academic.oup.com/nar/article-lookup/doi/10.1093/nar/16.22.10881). Green, GSH binding residues (g); cyan, typical ABC protein motifs: Walker A (a), Walker B (b), signature motif (s). Abbreviations: Cryneo, *Cryptococcus neoformans* JEC21; Crygat, *Cryptococcus gattii*; Saccer, *Saccharomyces cerevisiae*; Homsap, *Homo sapiens*; Musmus, *Mus musculus*; Aratha, *Arabidopsis thaliana*; Consen, consensus sequence. Download FIG S1, TIF file, 0.3 MB. Copyright © 2017 Garcia-Santamarina et al. 2017 Garcia-Santamarina et al. This content is distributed under the terms of the Creative Commons Attribution 4.0 International license.

Atm1 homologues are present in all eukaryotes and in some bacteria, with ~50% sequence identity among homologues from bacteria to humans (33). Multiple-sequence alignment performed with hierarchical clustering showed that this conservation is maintained within *Cryptococcus* spp. (Fig. S1B). We investigated whether *S. cerevisiae* Atm1 (*Sc*Atm1) expression is also induced under conditions of Cu stress in a fashion similar to that seen with *C. neoformans* Atm1 (*Cn*Atm1). Interestingly, *S. cerevisiae* cells treated with Cu showed no altered Atm1 expression (Fig. 1D), suggesting that the different organisms have intrinsically different ways to respond to Cu stress through Cu-regulated gene expression.

### *C. neoformans* Atm1 functions in cytosolic iron-sulfur protein formation.

*Sc*Atm1 functions by exporting an Fe-S cluster precursor from the mitochondrial matrix to the cytosol, which is used by the CIA machinery for the synthesis and maturation of cytosolic and nuclear Fe-S proteins (29, 30, 32). *S. cerevisiae* cells depleted of Atm1 have reduced growth rates and reduced activities of cytosolic and nuclear Fe-S cluster proteins and constitutively express a set of genes largely overlapping with the transcriptional response of yeast to Fe deficiency (34). As a consequence of the latter, *S. cerevisiae* cells lacking Atm1 accumulate iron in the mitochondria and have reduced levels of holo-heme proteins and increased levels of oxidative stress (30). To assess whether *Cn*Atm1 functions as a bona fide Atm1, *Cn*Atm1 was tested for complementation in *S. cerevisiae* strain GalL-Atm1, in which *ATM1* gene expression is under the control of the galactose-inducible and glucose-repressible *GALL* promoter. This strain was transformed with either an empty vector or a vector expressing *S. cerevisiae ATM1* or *C. neoformans ATM1*. As shown in Fig. S2A, *Cn*Atm1 rescued the growth of *S. cerevisiae* cells lacking Atm1 to an extent similar to that seen with cells expressing *Sc*Atm1. Similarly, the activity of the cytosolic Fe-S enzyme isopropyl malate dehydratase (Leu1) (Fig. S2B, left panel) was restored by expression of both *Sc*Atm1 and *Cn*Atm1, suggesting that the *C. neoformans* Atm1 protein is able to support the maturation of cytosolic Fe-S proteins. Moreover, *S. cerevisiae GalL-Atm1* cells depleted of endogenous *Sc*Atm1 and expressing *Cn*Atm1 increased activity of the heme-containing protein catalase compared to the results seen with the empty vector control, with no impact on a control enzyme (Fig. S2B, middle panel). As cells lacking Atm1 display disturbed iron homeostasis and increased the expression of the Aft1/Aft2 transcription factor-dependent iron regulon, we measured the expression of a reporter gene that is responsive to Fe deficiency (35, 36). Expression of both *Sc*Atm1 and *Cn*Atm1 in *S. cerevisiae* cells depleted of Atm1 severely decreased the activation of the Fe deficiency-responsive promoter (Fig. S2C).

10.1128/mBio.01742-17.3 FIG S2  *C. neoformans* Atm1 is a functional ortholog of *S. cerevisiae* Atm1. (A and B) The *S. cerevisiae* GalL-*ATM1* strain was transformed with empty vector or with a vector expressing *S. cerevisiae* Atm1 (*Sc*Atm1) or *C. neoformans* Atm1 (*Cn*Atm1) as indicated. Yeast cells were grown for 64 h on glucose medium. (A) Cells were spotted on minimal medium agar plates with 2% galactose or 2% glucose as indicated. Plates were incubated at 30°C for 3 days. (B) Cell extracts were assayed for specific activities of Leu1 and catalase, and values were normalized to those of malate dehydrogenase (MDH). For Leu1, *n* = 6 (one-way repeated-measures ANOVA test, *P* = 0.0007); for catalase, *n* = 6 (*P* < 0.0001); for MDH, *n* = 6 (*P* = 0.9). (C) *S. cerevisiae* GalL-ATM1 cells carrying the pAFT1-FLAG-lux reporter plasmid were transformed with empty vector or with a vector expressing *S. cerevisiae* Atm1 (*Sc*Atm1) or *C. neoformans* Atm1 (*Cn*Atm1) as indicated. Yeast cells were grown for 64 h on glucose medium. During the last 12 h of growth, the medium was supplemented with 50 µM FeCl_2_. Luciferase activities were determined in cell lysates and normalized to protein concentration. Bathophenanthroline disulfonic acid (BPS) (50 µM) was added to cells with empty vector to deplete iron and maximally induce the reporter gene. *n* = 6 (one-way repeated-measures ANOVA test, *P* < 0.0001). (D) The *C. neoformans GAL7* promoter allows Cuf1-independent and Cu-independent regulation of Atm1 expression. Cultures of strains Atm1-F (DTY947), Gal7-Atm1-F (#1; DTY949), and Gal7-Atm1-F (#2; DTY950) were back-diluted and grown for 3 days in SC-Gal media (left panel) or SC-Gluc media (middle panel). At day 3, cultures were diluted to an OD of 0.3 and grown for 2 h, and gene expression analysis was performed as described for Fig. 1A. For the left and middle panels, *n* = 3 (one-way repeated-measures ANOVA test, *P* = 0.001 and *P* = 0.01, respectively). For the right panel, cellular protein extracts from the cultures represented in the left and middle panels were analyzed by immunoblotting with FLAG and anti-histone 3 (H3; loading control) antibodies. Download FIG S2, TIF file, 0.1 MB. Copyright © 2017 Garcia-Santamarina et al. 2017 Garcia-Santamarina et al. This content is distributed under the terms of the Creative Commons Attribution 4.0 International license.

**FIG 2:**
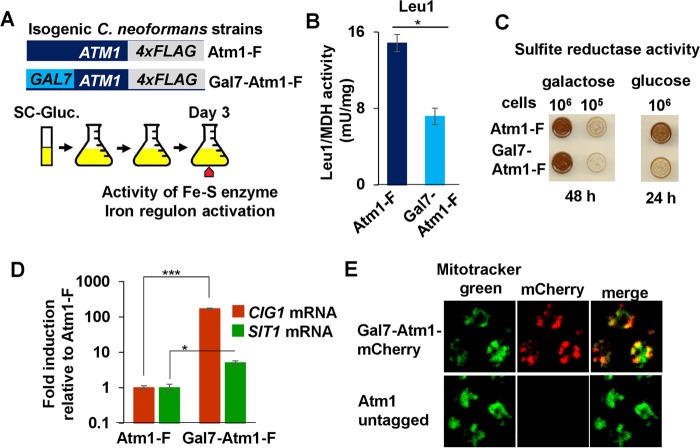
*C. neoformans* Atm1 is a functional ortholog of *S. cerevisiae* Atm1. (A) Scheme representing the steps for the experimental procedures performed as described for panels B to D. (B to D) Cultures of *C. neoformans* strains Atm1-F (DTY947) and Gal7-Atm1-F (DTY949) were back diluted in SC-Gluc media for 3 days. Cultures were diluted to the desired OD in SC-Gluc media and grown for 3 h, and the ratio of Leu1 (Fe-S enzyme) enzymatic activity to malate dehydrogenase (MDH) (lacking Fe-S clusters) enzymatic activity (B), the sulfite reductase activity (C), and the gene expression of *CIG1* and *SIT1* normalized to *ACT1* (D) were assayed. For Leu1/MDH activity analysis, *n* = 3 (Student’s *t* test of paired samples, *P* = 0.02). For *CIG1*/*ACT1* expression analysis, *n* = 3 (Student’s *t* test of paired samples, *P* = 0.0007). For *SIT1*/*ACT1* expression analysis, *n* = 3 (Student’s *t* test of paired samples, *P* = 0.02). (E) *C. neoformans* Atm1 localizes to mitochondria. Strains Gal7-Atm1-mCherry (DTY951) and H99 (Atm1-untagged) were grown in SC-Gal media for 3 days and incubated with Mitotracker green (for mitochondrial staining) and Atm1-mCherry localization. Samples were analyzed by confocal microscopy.

We tested whether *C. neoformans* cells lacking Atm1 show phenotypes similar to those seen with the Atm1-deficient *S. cerevisiae* strain. The native promoter of the *C. neoformans* epitope-tagged Atm1-F allele was replaced with the *GAL7* promoter (Gal7-Atm1-F) (Fig. 2A). We used the *GAL7* promoter instead of generating a *ΔAtm1* strain to subvert the Cu-dependent and Cuf1-dependent expression of *ATM1* and to prevent permanent strain adaptations to the lack of Atm1, which is required for the maturation of cytosolic and nuclear Fe-S proteins, some of which are essential. As confirmed by qRT-PCR and by immunoblotting (Fig. S2D), the Gal7-Atm1-F strain showed high levels of *ATM1-F* transcript and protein in galactose and reduced levels in glucose compared to the expression of *ATM1-F* under the control of its native promoter. After the isogenic strains were grown for 3 days in glucose, the activities of the cytosolic Fe-S proteins Leu1 (Fig. 2B) and sulfite reductase (Fig. 2C) were significantly decreased in the Gal7-Atm1-F strain upon depletion of Atm1. Similarly, two *C. neoformans* genes, *CIG1* and *SIT1*, previously shown to be activated during Fe starvation (37), showed increased expression in the Gal7-Atm1-F strain compared to the Atm1-F strain (Fig. 2D). Since *S. cerevisiae* Atm1 localizes to the mitochondrial inner membrane (38), we determined the localization of a *Cn*Atm1-mCherry fusion protein. Confocal microscopy revealed colocalization of Atm1-mCherry with Mitotracker green, demonstrating that *C. neoformans* Atm1 is a mitochondrial protein (Fig. 2E, merge). Together, these results demonstrate that *C. neoformans* Atm1 is a bona fide functional orthologue of *S. cerevisiae* Atm1.

### *C. neoformans* Atm1-depleted cells are more susceptible to Cu toxicity.

**FIG 3:**
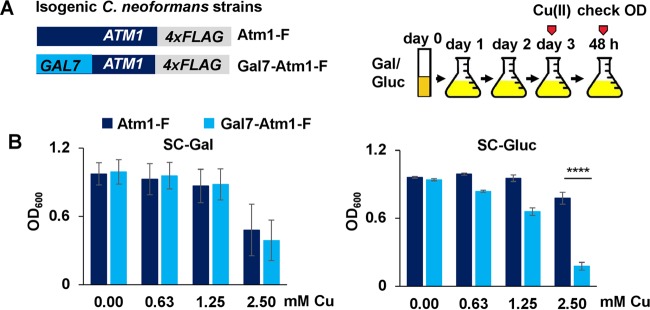
*C. neoformans* cells expressing low Atm1 protein levels are more sensitive to Cu stress than wild-type cells. (A) Scheme representing steps for the experimental procedures performed as described for panel B. Cultures of *C. neoformans* strains Atm1-F (DTY947) and Gal7-Atm1-F (DTY949) were diluted in either galactose-containing media or glucose-containing media for 3 days and diluted into 96-well plates to an OD of 0.002 in galactose-containing media or glucose-containing media, respectively, with the indicated concentrations of Cu. The final OD was recorded after 48 h. (B) Cultures of *C. neoformans* strains prepared as shown in panel A were grown in SC-Gal media (left panel) or in SC-Gluc media (right panel) with the indicated amounts of Cu. *n* = 3 (3-way repeated-measures ANOVA test). Cu differentially impacted growth of the strains only in the presence of glucose (*P* < 0.0001).

In *C. neoformans*, Cuf1-dependent activation of metallothionein genes *MT1* and *MT2* in response to Cu stress is crucial for survival within the host lungs, the primary site of infection (9). Since Atm1 expression is also activated in a Cuf1-dependent manner during Cu stress, we ascertained whether Atm1 is important for cell survival under conditions of high Cu concentrations. The Atm1-F and Gal7-Atm1-F strains were grown for 3 days either in galactose or glucose, diluted into 96-well plates, and grown with or without additional Cu (Fig. 3A). *C. neoformans* cells lacking Atm1 (Gal7-Atm1-F cells grown in glucose) exhibited a growth defect in the presence of Cu compared with cells expressing Atm1 under the control of the native promoter (Atm1-F) (Fig. 3B, right panel). This growth defect was not observed when the strains were grown in galactose as a carbon source (Fig. 3B, left panel). These results could be explained by decreased expression of the *MT1*/*2* genes upon Atm1 depletion. However, as shown in Fig. S3A, the *MT1*/*2* genes are induced several-hundred-fold, independently of Atm1 expression. *S. cerevisiae ΔAtm1* cells have a severe growth defect. On the basis of the functional similarity of Atm1 proteins from the two yeasts, we anticipated that Atm1-depleted *C. neoformans* cells would display a similar growth phenotype. Interestingly, even though the Atm1 protein was undetectable by immunoblotting in the Gal-Atm1-F strain after growth in glucose (Fig. S2D), this strain displayed only a slight growth defect, and that growth defect was no longer significant after 48 h, compared with the results seen with the Atm1-F strain in the absence of Cu stress. This phenotype could be attributed to a requirement for only low levels of Atm1 protein or to a compensatory function of another mitochondrial ABC transporter, Mdl1, both of which are effects previously described for *S. cerevisiae* (30, 33, 39).

10.1128/mBio.01742-17.4 FIG S3  *C. neoformans* cells expressing low Atm1 protein levels are more sensitive to Cu stress than wild-type cells. (A) *C. neoformans* cells with reduced Atm1 protein levels had *MT1*/*2* transcriptional responses to Cu stress similar to those seen with wild-type cells. Exponentially growing cultures of strains Atm1-F (DTY947) and Gal7-Atm1-F (DTY949) at day 3 of glucose growth, as described in Fig. 2E, were incubated without or with 2.5 mM Cu for 30 min. Total RNA was isolated, cDNA was synthesized, and gene expression analysis was performed with specific primers (see Table S2 in Text S1) for *MT1*, *MT2*, and *ACT1* (used for data normalization). *n* = 3 (3-way repeated-measures ANOVA test). *MT* gene expression results were not significantly different between the genotypes (for *MT1*, *P* = 0.2; for *MT2*, *P* = 0.14). (B) Cultures of *C. neoformans* prepared as described for panel Fig. 3A were grown in YNB-gal (left panel) or in YNB-gluc (right panel) with the indicated amounts of Cu. Cu differentially impacted the growth of the strains only in the presence of glucose (*P* < 0.0001). (C) *C. neoformans* strains *ΔMt1 ΔMt2* (DTY756) and *ΔMt1 ΔMt2* Gal7-Atm1-F (DTY953) were grown in SC-Gal medium (left panel) or in SC-Gluc medium (right panel) as described for panel Fig. 3A with the indicated amounts of Cu. *n* = 3 (3-way repeated-measures ANOVA test). Cu differently impacts the growth of the strains only in the presence of glucose (*P* < 0.0001). Download FIG S3, TIF file, 0.1 MB. Copyright © 2017 Garcia-Santamarina et al. 2017 Garcia-Santamarina et al. This content is distributed under the terms of the Creative Commons Attribution 4.0 International license.

Components of yeast media, such as the amino acids cysteine, methionine, and histidine, along with other thiol-containing compounds, can chelate Cu (40). Since *C. neoformans* grows in minimal media lacking these nutrients, we ascertained whether the Cu sensitivity phenotype shown by Atm1-depleted cells would be enhanced if the cells were grown in a medium with lower levels of Cu chelation properties. As shown in Fig. S3B, right panel, the Gal7-Atm1-F strain grown in glucose medium (i.e., with undetectable Atm1 expression) and in the absence of exogenous amino acids was about 30-fold more sensitive to Cu stress than the same strain grown in the presence of amino acids (Fig. S3B, right panel), suggesting that Cu buffering is important in preventing Cu toxicity in the absence of Atm1. To test whether the role of Atm1 in Cu toxicity would be additive with respect to that of the metallothioneins, the *GAL7-ATM1* allele was placed into a *C. neoformans ΔMt1 ΔMt2* strain (*ΔMt1 ΔMt2* Gal7-Atm1). The *ΔMt1 ΔMt2* and *ΔMt1 ΔMt2* Gal7-Atm1 strains were grown in galactose or glucose and in the presence or absence of Cu stress. Atm1-depleted cells (Fig. S3C) showed significantly reduced growth in the presence of Cu compared to cells expressing Atm1 from the native promoter, with increased Cu sensitivity of the *ΔMt1 ΔMt2* Gal7-Atm1 strain to Cu compared to a Gal7-Atm1-F strain (compare Fig. 3B to S3C). This suggests that the role of Atm1 in preventing Cu toxicity is complementary and additive with respect to the Cu sequestration function of the *C. neoformans* metallothioneins.

### Fe-S proteins are targets of Cu toxicity in *S. cerevisiae.*

**FIG 4:**
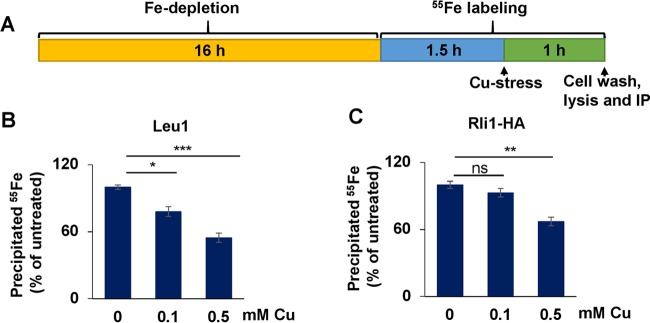
Cu targets cytosolic solvent-exposed Fe-S clusters. (A) Experimental setup for ^55^Fe labeling experiments. Cells were grown for 16 h in Fe-poor SC medium with glucose and radiolabeled with 10 µCi ^55^Fe for 1.5 h, CuSO_4_ was added, and cells were grown in the presence of both ^55^Fe and Cu for 1 h. (B) Wild-type (WT) *S. cerevisiae* cells were grown as described for panel A. Endogenous Leu1 protein was immunoprecipitated from cell extracts with specific antibodies. The amount of coprecipitated ^55^Fe was quantified by scintillation counting. Data are presented relative to the values obtained for samples not treated with Cu. *n* = 8 (one-way repeated-measures ANOVA test, *P* < 0.0001). (C) WT cells transformed with vector overproducing Rli1-HA were grown as described for panel A and processed as described for panel B. *n* = 8 (*P* = 0.005).

Previous studies have implicated Fe-S proteins among the targets of Cu toxicity. These studies showed that Cu displaces Fe ions from solvent-exposed Fe-S clusters, suggesting that Cu(I) damages Fe-S proteins by directly binding to the coordinating sulfur atoms (24, 26–28). Thus, we sought to determine if Cu might also damage Fe-S clusters in *S. cerevisiae* using an *in vivo* ^55^Fe incorporation method for detection of Fe-S clusters (Fig. 4A). Wild-type cells were incubated in Fe-poor medium for 16 h to deplete cells of Fe and to allow boosting of ^55^Fe-S cluster incorporation into apoproteins. After an initial 1.5 h of ^55^Fe labeling, cells were incubated with Cu for 1 h, washed, and lysed, and clarified lysates were subjected to immunoprecipitation performed with specific antibodies. The precipitated ^55^Fe radioactivity was quantified by scintillation counting.

We first tested whether two cytosolic Fe-S proteins, Leu1 and Rli1, were affected by Cu treatment. While structural studies demonstrated that Leu1 possesses a solvent-exposed [4Fe-4S] cluster (41), the two [4Fe-4S] clusters of the essential protein Rli1 were occluded from solvent (42, 43). In both cases, there was a significant decrease in the ^55^Fe counts in the presence of Cu, in a dose-dependent fashion, with no change in steady-state protein levels (Fig. 4B and C and S4A and B). Significantly, while incubation of *S. cerevisiae* cells with 0.1 mM Cu was sufficient to produce a significant impact on ^55^Fe-S associated with Leu1 (Fig. 4B), 0.5 mM Cu was required for a similar diminution of the Rli1 Fe-S clusters (Fig. 4C), suggesting that Cu was more effectively targeting solvent-exposed Fe-S clusters. Similarly, the Leu1 enzymatic activity was reduced by ~50% after 2.5 h of stress performed with 1.25 mM Cu (Fig. S4C). As mitochondria represent the primary site of synthesis of the Fe-S cluster precursor exported by Atm1 (35), we tested the effects of Cu stress on mitochondrial Fe-S proteins. The [4Fe-4S] cluster in aconitase (44) (Fig. S4D, left panel) and the [2Fe-2S] cluster of ectopically expressed human ferredoxin (45) (Fig. S4E, left panel), both solvent exposed, required 0.5 mM Cu to produce a weak and yet significant decrease in the level of the protein-associated ^55^Fe-S cluster. The aconitase activity was affected only minimally after exposure to 1.25 mM Cu stress for either 2.5 h or 16 h (Fig. S4D, middle and right panels). Moreover, the Fe-S cluster of endogenous mitochondrial ferredoxin Yah1 was not affected by Cu stress at the concentrations of the metal tested (Fig. S4E, right panel). This is in contrast with the results from a recent publication in which ^55^Fe incorporation into the Yah1 Fe-S cluster was reported to have been significantly diminished after 1 mM Cu treatment (46). The disparity could be attributed to differences in the ^55^Fe immunoprecipitation experiments. We usually observe amounts of ^55^Fe associated with immunoprecipitated Yah1 (or other Fe-S cluster binding proteins) that are at least 100-fold lower than the published data (46). Moreover, a disruption of the Yah1 Fe-S cluster should be accompanied by a lack of Fe-S clusters associated with Isu1 (47), Nar1, or Rli1; no such result was seen in the previous report (46). Collectively, our results suggest that mitochondrial Fe-S proteins are more resistant to Cu toxicity than those in the cytoplasm or nucleus.

10.1128/mBio.01742-17.5 FIG S4  Mitochondrial Fe-S proteins are partially protected from Cu stress. (A and B) Protein levels of Leu1 (A) (from the experiment described in the Fig. 4B legend) and Rli1 (B) (from the experiment described in the Fig. 4C legend) were determined by immunoblotting. Porin served as a loading control. (C) Leu1/MDH activity in extracts of wild-type *S. cerevisiae* cells that were either left untreated or treated with 1.25 mM Cu for 2.5 h (*t* test paired samples, *n* = 4, *P* = 0.002). (D) (Left panel) *S. cerevisiae* WT cells were grown as described for Fig. 4A. Aconitase was immunoprecipitated from cell extracts with specific antibodies. The amount of coprecipitated ^55^Fe was quantified by scintillation counting. Data are presented relative to the values obtained for samples not treated with Cu. Protein levels in the indicated strains were determined by immunostaining. Porin and Hsp70 served as a loading control. *n* = 8. (Middle and right panels) Aconitase/MDH activity was measured in *S. cerevisiae* WT cell extracts before and after 2.5 h (middle panel) or 16 h (right panel) of 1.25 mM Cu stress. For both panels, *n* = 4 and *P* = ns. (E) (Left panel) *S. cerevisiae* WT cells transformed with a vector overproducing human ferredoxin (FDX2-HA) were grown as described for Fig. 4A and processed as described for panel A. Left panel, *n* = 8 (*P* = 0.0006). Right panel, *S. cerevisiae* cells were grown as described for Fig. 4A and processed as described for panel A. Left panel, *n* = 2 (*P* = ns). Download FIG S4, TIF file, 0.1 MB. Copyright © 2017 Garcia-Santamarina et al. 2017 Garcia-Santamarina et al. This content is distributed under the terms of the Creative Commons Attribution 4.0 International license.

**FIG 5:**
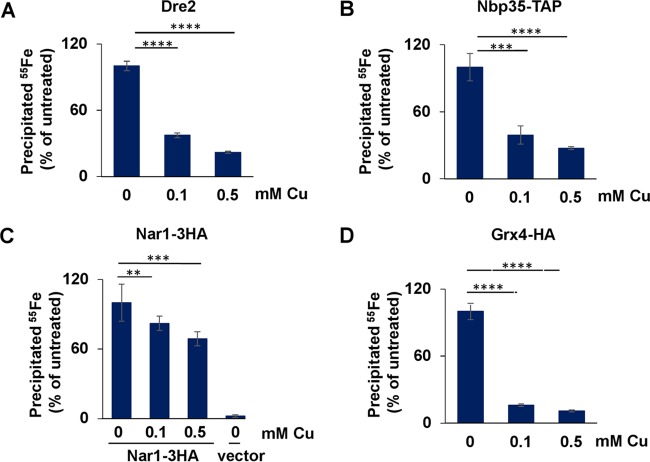
Critical targets for Cu toxicity are Fe-S clusters of proteins with functions in the CIA machinery and iron regulon activation. (A to D) WT *S. cerevisiae* cells (A), WT cells transformed with vector overproducing Nbp35-TAP (B) or Nar1-3HA (C), and WT cells in which the *GRX4* gene was genomically tagged with the HA epitope (D) were grown as described for Fig. 4A. The overproduced Nbp35-TAP and Nar1-3HA or endogenous Dre2 and Grx4-HA proteins were immunoprecipitated from cell extracts with specific antibodies. The amount of coprecipitated ^55^Fe was quantified by scintillation counting. Data are presented relative to the values obtained for samples not treated with Cu. *n* = 6 for Nar1 and *n* = 8 for the remaining proteins (one-way repeated-measures ANOVA test, *P* < 0.0001 for Dre2, Nbp35, Nar1, and Grx4).

As stronger defects were observed for cytosolic solvent-exposed Fe-S proteins than for mitochondrial Fe-S proteins, we investigated whether Cu could affect Fe-S clusters bound to CIA components (either transiently bound for subsequent insertion onto client apoproteins or permanently bound and required for function). If Cu disrupts Fe-S clusters in such CIA proteins, this may cause a decline in the assembly of cytosolic and nuclear apoproteins (48). In testing ^55^Fe incorporation into the CIA protein Dre2 (49) (Fig. 5A) and the Fe-S scaffold protein Nbp35 (50) (Fig. 5B), we unexpectedly observed that both proteins were significantly more severely affected than the cytosolic client proteins Leu1 and Rli1 (compare to Fig. 4). In contrast, the Fe-S clusters of Nar1 (51) (Fig. 5C) were more stable than those of Dre2 and Nbp35. The concentrations of all of those proteins did not change during the course of the treatment (Fig. S5A to C). Precise structural information on these CIA proteins is still missing, but the Fe-S clusters are expected to be solvent accessible in Dre2 and Nbp35, and yet Nar1 may contain both a solvent-exposed cluster and a buried cluster (51).

10.1128/mBio.01742-17.6 FIG S5  Cu stress during or after ^55^Fe labeling does not change the impact on cytosolic and nuclear Fe-S proteins. (A to D) Protein levels of strains described in the Fig. 5 legends as indicated were determined by immunoblotting. Porin and Hsp70 served as a loading control. (E) Experimental setup for ^55^Fe labeling experiments. Cells were grown for 14 h in Fe-poor SC medium. CuSO_4_ was added for 2 h in the same Fe-poor SC medium, and cells were radiolabeled with 10 µCi ^55^Fe in the presence of CuSO_4_ for 2 h. (F) (Left panel) Wild-type (WT) *S. cerevisiae* cells were grown as described for panel E. Endogenous Leu1 protein was immunoprecipitated from cell extracts with specific antibodies. The amount of coprecipitated ^55^Fe was quantified by scintillation counting. Data are presented relative to the values obtained for samples not treated with Cu. *n* = 2 (*P* = 0.02). (Right panel) Leu1/MDH activity was measured in extracts from WT *S. cerevisiae* cells before or after 16 h of 1.25 mM Cu stress. Data are presented as percentages of the activity of untreated samples. *n* = 4 (*P* = 0.002). (G) WT cells transformed with vector overproducing Rli1-HA were grown as described for panel E and processed as described for panel F, left panel. *n* = 2 (*P* = 0.2). (H) WT cells were grown overnight in SD medium. Cu stress was applied for 1 h (final concentration of 0.1 mM or 0.5 mM as indicated). Protein levels of Isu1 were determined by immunostaining of cell extracts. Porin served as a loading control. Download FIG S5, TIF file, 0.1 MB. Copyright © 2017 Garcia-Santamarina et al. 2017 Garcia-Santamarina et al. This content is distributed under the terms of the Creative Commons Attribution 4.0 International license.

In order to delve into the mechanism accounting for the fact that the Fe-S clusters from cytosolic CIA client proteins were more stable than those from the CIA machinery, we investigated Fe-S cluster stability in Leu1 and Rli1 when the ^55^Fe incorporation was performed in the presence of Cu. We envisioned that if Cu were present at the time of Fe-S cluster assembly, this would have a higher impact on cytosolic and nuclear targets. Thus, after 2 h of initial Cu stress, cells were labeled with ^55^Fe during 2 h together with the presence of Cu (i.e., 4 h of Cu stress in total) before cell washing, lysis, immunoprecipitation with specific antibodies, and counting of immunoprecipitated ^55^Fe were performed (Fig. S5E). Unexpectedly, we observed an impact on ^55^Fe-S associated with Leu1 (Fig. S5F, left panel) and Rli1 (Fig. S5G) similar to that seen when proteins were preloaded with a ^55^Fe-S cluster before Cu stress. Similarly, the decrease in enzymatic activity of Leu1 (Fig. S5F, right panel) seen upon 2.5 h of Cu stress was not further exacerbated by longer (16-h) Cu exposure (compare Fig. 4B and C). Surprisingly, the level of Fe-S biosynthetic activity of the CIA machinery was still high enough to partially assemble cytosolic Fe-S proteins in the presence of Cu. This is in agreement with previous *in vitro* experiments showing that, independently of transfer of Fe-S clusters through protein-protein interactions, the presence of excess Cu results in displacement of Fe from those Fe-S clusters for which it has higher binding affinity (28).

Finally, the effects of Cu stress on the Fe-S cluster of the cytosolic monothiol glutaredoxin Grx4 was evaluated (Fig. 5D). Grx4 has established roles in maintaining iron homeostasis in the cell and in assisting cytosolic-nuclear Fe-S protein biogenesis (35, 52). Grx4 possesses a solvent-exposed, bridging glutathione (GSH)-coordinated [2Fe-2S] cluster (53). Treatment with 0.1 mM Cu resulted in a pronounced decrease in ^55^Fe binding (Fig. 5D) without any change in the protein concentration (Fig. S5D). When the Grx4 Fe-S site is unoccupied, this leads to activation of the iron regulon via the Aft1/2 transcription factors (54, 55). Surprisingly, although Fe binding by Grx4 was strongly decreased upon Cu treatment, the iron regulon was not activated, even when the cells were treated with Cu overnight (data not shown). How this happens will require further exploration, but cells could be implementing a mechanism to prevent additional oxidative stress that would be caused by Fe influx. As a further test of the functional state of the Fe-S protein biogenesis machinery, we evaluated Isu1 protein levels, which are elevated by protein stabilization even during slight perturbations of the ISC machinery (56, 57) (M. A. Uzarska, unpublished observation). We observed an increase in Isu1 levels at Cu concentrations that destabilize solvent-exposed Fe-S clusters (Fig. S5H), further suggesting that Cu perturbs the function of the Fe-S protein assembly machinery. Taken together, in agreement with studies in bacteria, these results suggest that Cu differentially affects the stability and/or assembly of Fe-S proteins in eukaryotic cells. Cytosolic Fe-S proteins are more sensitive to Cu than mitochondrial ones, especially the Fe-S cluster-containing CIA proteins and Grx4, even though they maintain partial efficiency with respect to assembly of cytosolic Fe-S proteins.

### *C. neoformans* Fe-S cluster homeostasis during Cu stress.

**FIG 6:**
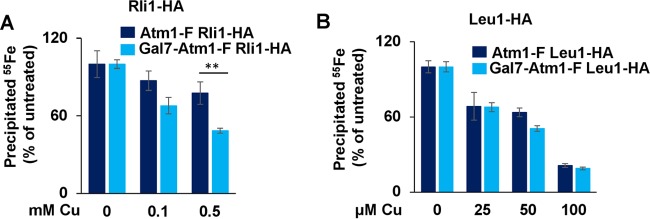
*C. neoformans* Atm1 contributes to the maintenance of cytosolic Fe-S cluster homeostasis during Cu stress. (A) Atm1-F Rli1-HA (DTY955) and Gal7-Atm1-F Rli1-HA (DTY957) *C. neoformans* cells were grown for 88 h in SC-Gluc medium to deplete Atm1 protein levels. During the last 16 h, cells were grown in Fe-poor SC-Gluc medium, and cells were radiolabeled with 10 µCi ^55^Fe for 1.5 h before Cu was added and cells were incubated in the presence of both ^55^Fe and Cu for 1 h (see Fig. 4A). Rli1-HA protein was immunoprecipitated from cell extracts with specific antibodies. The amount of coprecipitated ^55^Fe was quantified by scintillation counting. Data are presented relative to the values obtained for samples not treated with Cu. *n* = 4 (two-way repeated-measures ANOVA test, *P* = 0.04). (B) Atm1-F Leu1-HA (DTY959) and Gal7-Atm1-F Leu1-HA (DTY961) *C. neoformans* cells were experimentally processed and analyzed as described for panel A. *n* =4 (two-way repeated-measures ANOVA test, *P* = 0.1).

Given that *C. neoformans* Atm1 is a functional orthologue of *S. cerevisiae* Atm1, the Cu-induced Cuf1-dependent activation of *C. neoformans* Atm1 expression could be a mechanism aimed at maintaining cytosolic and nuclear Fe-S cluster homeostasis during Cu stress. To address this hypothesis, we first measured ^55^Fe incorporation into the *C. neoformans* Rli1 homologue fused to the hemagglutinin (HA) epitope tag in the Atm1-F- and Atm1-depleted Gal7-Atm1-F strains in the presence or absence of Cu stress, according to the scheme shown in Fig. 4A. As shown in Fig. 6A, levels of ^55^Fe associated with Rli1 decreased with increasing Cu concentrations, while Rli1 protein levels remained unaltered in both strain backgrounds (Fig. S6A). Rli1 is among the most highly conserved proteins in nature, with two [4Fe-4S] clusters occluded from the solvent (42, 43). On the basis of published results from Cu toxicity experiments performed to analyze proteins with buried Fe-S clusters *in vitro* (24, 28) and of our data in Fig. 4C, we predicted that it would be difficult for Cu to directly target the Rli1 Fe-S clusters, and the decrease of the level of ^55^Fe associated with Rli1 might more likely reflect the effect of the presence of functionally compromised CIA machinery during Cu stress. Atm1 depletion slightly enhanced the loss of ^55^Fe in Rli1-HA upon Cu stress compared with the isogenic strain with wild-type Atm1 protein levels (Fig. 6A). However, Atm1 depletion did not influence the amount of ^55^Fe associated with Rli1 in an experiment in which Cu stress was applied before ^55^Fe-S cluster assembly as schematized in Fig. S5E (Fig. 6SB), rendering a direct function of Atm1 in protecting Rli1 Fe-S cluster loading unlikely.

10.1128/mBio.01742-17.7 FIG S6  *C. neoformans* Atm1 contributes to the maintenance of cytosolic Fe-S cluster homeostasis during Cu stress. (A) Levels of protein from the strains described in the Fig. 6A legend were determined by immunostaining. Porin served as a loading control. (B) Atm1-F Rli1-HA (DTY955) and Gal7-Atm1-F Rli1-HA (DTY957) *C. neoformans* cells were grown for 88 h in SC-Gluc medium in order to deplete Atm1 protein levels. For the last 22 h, cells were grown in Fe-poor SC-Gluc medium with 0.6 mM CuS0_4_. Cells were radiolabeled with 10 µCi ^55^Fe for 2 h in the presence of Cu (the total time with Cu was 24 h). Rli1-HA protein was immunoprecipitated from cell extracts with specific antibodies. The amount of coprecipitated ^55^Fe was quantified by scintillation counting. (C) Levels of proteins from the strains described in the Fig. 6B legend were determined by immunostaining. Porin served as a loading control. (D) Activities of Leu1/MDH were analyzed in extracts obtained from either untreated or Cu-treated (3 h) (as indicated) exponentially growing cultures of strains Atm1-F (DTY947) and Gal7-Atm1-F (DTY949) at day 3 after glucose depletion in YNB-Gluc medium, as described in the Fig. 2E legend. MDH activity was used for Leu1 activity normalization (not shown). Data were normalized to enzyme ratios obtained for cells grown without Cu. *n* = 4 (2-way repeated-measures ANOVA test, *P* = 0.48). Download FIG S6, TIF file, 0.1 MB. Copyright © 2017 Garcia-Santamarina et al. 2017 Garcia-Santamarina et al. This content is distributed under the terms of the Creative Commons Attribution 4.0 International license.

Along the same lines, although the levels of ^55^Fe associated with *C. neoformans* Leu1-HA decreased upon Cu stress whereas the protein levels remained unchanged (Fig. 6B and C), this decrease was unaffected by Atm1 depletion. The disruption of the Leu1 Fe-S cluster by Cu had an impact on Leu1 enzymatic activity, and yet this effect was not significantly exacerbated by a depletion of Atm1 (Fig. S6D).

The weak or absent influence of Atm1 on the protection of the Fe-S clusters of these two cytosolic proteins suggests that their Fe-S clusters are not responsible for the strong Cu effect on the growth of Atm1-deficient cells (cf. Fig. 3 and S3). We therefore speculate that Cu might affect other essential cytosolic and/or nuclear proteins that require Atm1 for function, such as the Fe-S cluster-containing DNA polymerases, primases, and ATP-dependent helicases or the iron protein ribonucleotide reductase (32, 58).

**FIG 7:**
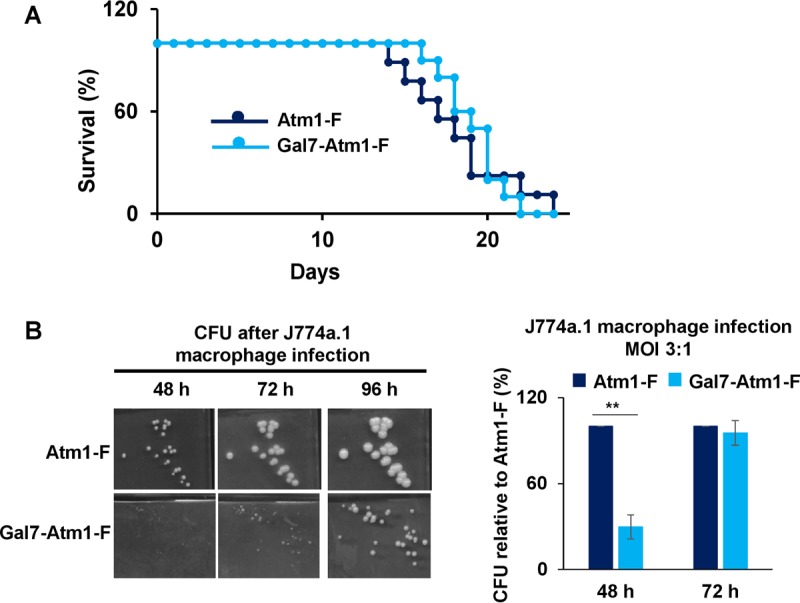
*C. neoformans* Atm1-depleted cells have decreased fitness upon coincubation with macrophage-like cell lines. (A) *C. neoformans* Atm1-depleted cells showed a trend toward reduced virulence in an intranasal mouse model of cryptococcosis. Cultures of strains Atm1-F (DTY967) and Gal7-Atm1-F (DTY969) were grown in SC-Gluc medium for 2 days and inoculated intranasally into two groups of 10 A/J mice with an inoculum size of 10^5^ cells per mouse. Survival data are represented in a Kaplan-Meier plot, and statistical analysis was performed with the log-rank test (*P* = 0.82). (B) Cultures of strains prepared as described for panel A were grown in SC-Gluc medium for 2 days, diluted to 5 × 10^5^ cells per ml in Dulbecco’s modified Eagle’s medium (DMEM), opsonized, and coincubated with the activated J774a.1 murine macrophage-like cell line at a multiplicity of infection (MOI) of 3:1. After the coincubation period, *C. neoformans* colonies were plated in SC-Gluc medium and representative pictures were taken at the indicated times (left panel). CFU counts were determined after 48 h or 72 h of incubation at 30°C and are presented relative to the data obtained for the Atm1-F strain (right panel). *n* = 3 (two-way repeated-measures ANOVA test, *P* = 0.03).

We tested whether the toxic effect exerted by Cu on Atm1-depleted cells could be potentiated using zinc-pyrithione (ZPT), a small-molecule Cu ionophore that has potent antifungal properties and which was previously shown to cause damage to Fe-S clusters in proteins as part of its mechanism of action (59, 60). ZPT is an ionophore that rapidly exchanges the Zn(II) with a Cu(II) ion for which it has higher affinity. The mechanism of Cu-pyrithione action is not fully understood, but, potentially, it releases Cu within the cell, as supported by the ability of this compound to potently activate the Cu regulon (59, 60) (Fig. S7A). To test the toxicity of ZPT in *C. neoformans* cells depleted for Atm1, Atm1-F and Gal7-Atm1-F strains were grown in galactose or glucose, diluted into 96-well plates, and treated with increasing concentrations of ZPT (Fig. S7B). While there was no discernible difference in the growth rates of cells expressing Atm1 from the native promoter and of cells expressing Atm1 from the Gal7 promoter on synthetic complete galactose medium (SC-Gal) (Fig. S7A), Atm1-depleted cells did not grow in concentrations as low as 0.01 μM ZPT, a concentration at least 100-fold lower than that required for preventing the growth of cells expressing Atm1 from the native promoter (Fig. 7B; SC-Gluc). We ascertained whether the growth differences between Atm1-F and Gal7-Atm1-F on glucose medium were accompanied by differences in the activities of Fe-S proteins. Leu1 activity was tested after treatment of cells with 0.1 μM ZPT in the presence of 10 μM Cu. As shown in Fig. S7C, cells depleted of Atm1 had significantly reduced Leu1 activity upon ZPT and Cu treatment compared with the Leu1 activity seen in Atm1-F cells. Interestingly, Leu1-HA protein levels decreased in Atm1-F cells in a parallel experiment under conditions of treatment with ZPT and Cu (Fig. S7D), suggesting that the recovered Leu1 activity in Atm1-F cells after 2 and 4 h of treatment could have been due to an Fe-S cluster repair mechanism driven by Atm1. However, the decrease in Leu1 activity alone cannot explain the differences in lethality between Atm1-expressing cells and Atm1-deficient cells, suggesting that either an essential Fe-S protein or a combination of effects in several Fe-S proteins was being targeted by the Cu ionophore.

10.1128/mBio.01742-17.8 FIG S7  *C. neoformans* Atm1 contributes to the maintenance of cytosolic but not mitochondrial Fe-S cluster homeostasis during Cu-ionophore stress. (A) Scheme of potential zinc-pyrithione (ZPT) mechanism for providing intracellular bioavailable Cu in *C. neoformans*. Cu2^+^ ions have high pyrithione binding affinity and, when added to cells together with ZPT, may displace Zn^2+^ ions from the Zn-pyrithione complex. The newly formed Cu-pyrithione complex diffuses through the plasma membrane into the cytosol and other intracellular membranous organelles, including mitochondria. Cu complexed with pyrithione, Cu alone, or both provide a source of bioavailable intracellular Cu that can potentially impact intracellular Fe-S clusters. (B) Cultures of *C. neoformans* strains Atm1-F (DTY947) and Gal7-Atm1-F (DTY949) were grown in SC-Gal media (left panel) or in SC-Gluc media (right panel) for 3 days and diluted into 96-well plates to an OD of 0.002 in SC-Gal or SC-Gluc, respectively, with the indicated concentrations of ZPT. The final OD was recorded after 48 h. *n* = 3 (3-way repeated-measures ANOVA test). ZPT differentially impacted growth only in the presence of glucose (*P* < 0.0001). (C) Activities of Leu1/MDH were analyzed in protein extracts obtained from either untreated or ZPT/Cu-treated (as indicated) exponentially growing cultures of Atm1-F (DTY947) and Gal7-Atm1-F (DTY949) at day 3 after glucose depletion in SC-Gluc medium. MDH activity was used for Leu1 activity normalization. Data are plotted relative to the activities of ZPT/Cu-untreated samples for each strain. *n* = 4 (two-way repeated-measures ANOVA test, *P* = 0.1). (D) Cellular protein extracts from cultures of strains Atm1-F Leu1-HA (DTY959) (Atm1-F Leu1-HA) and Gal7-Atm1-F Leu1-HA (DTY961) grown as described for the right panel in Fig. 7, either left untreated or treated with 0.1 μM ZPT and 10 μM Cu for the indicated time periods, were analyzed by immunoblotting with HA, FLAG, and Cdc2 (loading control) antibodies. Download FIG S7, TIF file, 0.2 MB. Copyright © 2017 Garcia-Santamarina et al. 2017 Garcia-Santamarina et al. This content is distributed under the terms of the Creative Commons Attribution 4.0 International license.

### Low levels of Atm1 impact *C. neoformans* fitness in macrophages and mice.

The *C. neoformans* Cu-buffering metallothioneins are virulence factors in intranasal mouse models of cryptococcosis (9). On the basis of the observed growth defect of *C. neoformans* Atm1-depleted cells upon Cu exposure, we hypothesized that this Cu sensitivity could compromise *C. neoformans* growth during host colonization. To test the contribution of Cu-dependent expression of Atm1 to virulence, intranasal mouse infections were conducted with the Atm1-F and Gal7-Atm1-F strains after 2 days of Atm1 depletion in glucose. As shown in Fig. 7A, there was a modest, though not statistically significant, decrease in Gal7-Atm1-F strain virulence compared to that seen with the wild type. However, we could not assess whether Atm1 levels were maintained or repressed during the course of the *in vivo* infection in the *GAL7* promoter-controlled strain. Furthermore, these strains are still able to induce expression of *MT1*/*2*, which are by far the main factors contributing to Cu detoxification in this fungus.

In order to further assess the contribution of Atm1 to virulence, but in a more controlled system, we explored whether Atm1 depletion would affect growth of *C. neoformans* cells after macrophage phagocytosis. Host innate immune cells such as macrophages use Cu as a microbiocidal agent against microbial pathogens by accumulating and compartmentalizing the metal into the phagosome. Lipopolysaccharide (LPS)- and gamma interferon (IFN-γ)-activated macrophages coordinately increase the expression of both the high-affinity Cu^+^ importer Ctr1 at the plasma membrane and the Cu^+^-transporting P-type ATPase, ATP7A, at the phagolysosomal membrane (20). We used the J774a.1 macrophage-like cell line and infected it with *C. neoformans* Atm1-F and Gal7-Atm1-F strains, the latter depleted for Atm1 by growth in glucose for 2 days. J774a.1 cells were activated with LPS and IFN-γ for 24 h and then independently infected with the two *C. neoformans* strains opsonized with the monoclonal 18B7 antibody. After 2 h, nonphagocytosed *C. neoformans* cells were washed away and J774a.1 cells with phagocytosed *C. neoformans* cells were further incubated for an additional 24 h before lysing of macrophages and plating of fungal cells for measuring CFU levels were performed. As shown in Fig. S8, the two strains were equally phagocytosed by the J774a.1 cells. However, as shown in Fig. 7B, CFU recovered from the Gal7-Atm1-F strain represented a significant growth delay compared with CFU recovered from the Atm1-F strain. Even though the colony remained smaller for the Gal7-Atm1-F strain, the number of CFU matched that of the wild-type Atm1-F strain, detected after 72 h of incubation at 30°C. These results suggest that macrophages cause higher levels of stress in Atm1-depleted *C. neoformans* than in wild-type cells, with the consequences of this stress being fungistatic rather than fungicidal.

10.1128/mBio.01742-17.9 FIG S8  Lack of Atm1 does not impair the uptake of *C. neoformans* into macrophages. Cultures of strains grown as described for Fig. 7B were serially diluted in SC-Gluc medium for two consecutive days. At day 2, cells were diluted to 5 × 10^5^ cells per ml in DMEM and coincubated with the J774a.1 murine macrophage-like cell line for 2 h. Nonphagocytosed *C. neoformans* cells were washed away, and after macrophages were lysed, released *C. neoformans* colonies were plated in SC-Gluc medium and counted after 48 h or 72 h at 30°C. The phagocytosis index was calculated as the ratio of CFU after 2 h of incubation to the number of CFU in the inoculum. Data were normalized (%) with the CFU counts obtained for the Atm1-F strain. *n* = 3 (two-way repeated-measures ANOVA test, *P* = 0.08). Download FIG S8, TIF file, 0.02 MB. Copyright © 2017 Garcia-Santamarina et al. 2017 Garcia-Santamarina et al. This content is distributed under the terms of the Creative Commons Attribution 4.0 International license.

## DISCUSSION

While the toxicity of Cu as an antimicrobial agent has been harnessed for centuries, our understanding of the mechanisms of Cu antimicrobial activity against cell growth is surprisingly poor. Given the role of Cu ionophores as antimicrobials, and the requirement for Cu for innate immune cell function in coping with infections, a better understanding of what causes Cu to be toxic to microorganisms could lead to the development of effective, broad-spectrum antimicrobials. Moreover, understanding fundamental aspects of Cu toxicity is likely to provide additional insights into the pathophysiology of human diseases of Cu overload such as Wilson’s disease.

Previous work demonstrating the importance of Cuf1, the Cu^+^ transporters, and metallothioneins in virulence in different host infectious niches supports a role for both Cu acquisition and Cu detoxification in the *C. neoformans* infectious journey (9, 10). While the Mt1 and Mt2 proteins are powerful Cu sequestration tools that *C. neoformans* uses for Cu detoxification, we demonstrate in this work that the Cuf1- and Cu-dependent activation of the mitochondrial ABC transporter Atm1 is a novel additional mechanism used by this organism to cope with Cu toxicity. Cells expressing Atm1 grew much better in the presence of increasing levels of Cu than cells in which Atm1 was downregulated (Fig. 3B, left and right panels). Previous studies in yeast, mouse, humans, and plants firmly established the structure and function of Atm1 as a critical exporter of a precursor in the Fe-S cluster biogenesis pathway (30, 35, 61). Here, we confirm the function of *C. neoformans* Atm1 in cytosolic Fe-S protein biogenesis.

While Rli1 with its two stably bound [4Fe-4S] clusters (62, 63) and the [4Fe-4S] hydratase Leu1 were differentially sensitive to increasing levels of Cu, the additional depletion of Atm1 did not consistently further diminish the Fe-S cluster content in these two proteins. This finding was independent of whether Fe-S cluster assembly was tested (Cu addition before the ^55^Fe assay) or whether the Fe-S cluster stability was analyzed (^55^Fe-S cluster generated before Cu addition). We therefore postulate that other Atm1-dependent cytosolic and/or nuclear Fe-S proteins or the iron protein ribonucleotide reductase (58) might be responsible for the strongly increased growth defect of Atm1-depleted cells seen in the presence of Cu. Fe-S candidate proteins are DNA polymerases and primases or the ATP-dependent DNA helicases (32). Hence, future studies need to identify and analyze these essential Fe-S proteins to ascertain which of them might be responsible for the growth defect of Atm1-depleted cells in the presence of toxic concentrations of Cu. Nevertheless, cytosolic solvent-exposed Fe-S clusters seem to be perturbed by Cu by a combination of mechanisms. First, the solvent-exposed environment allows Cu to directly disrupt the cofactor, similarly to the effect of Cu in the Fe-S clusters from bacterial dehydratases. We note, however, that Leu1 seems to be much less severely and less selectively affected than bacterial enzymes (24). Second, the striking functional impairment of the Fe-S proteins of the CIA machinery observed in our study may delay the *de novo* assembly of the cofactor, at least in some (still unknown) sensitive Fe-S proteins. This may be the reason that *C. neoformans* increases Atm1 expression as an attempt to maintain essential cellular functions that depend on Fe-S clusters.

We have shown in this study that one of mechanisms of Cu toxicity in fungi is alteration of Fe-S cluster homeostasis in the cell by direct disruption of cofactor binding to target proteins. Our detailed analysis of the effects of Cu on different cellular Fe-S proteins revealed that the most Cu-sensitive Fe-S proteins contain a transiently bound or solvent-exposed cluster. Unexpectedly, the effect of Cu stress on Fe-S cluster disruption was more pronounced in members of the CIA machinery than in two analyzed downstream clients. A plausible explanation could be inferred from *in vitro* data from a recent report (28). In that work, it was shown that Fe-S clusters can be transferred through protein-protein interactions to downstream Cu-bound targets. However, very interestingly, the higher thiophilicity of the released Cu seen upon Fe-S transfer would immediately disrupt the newly formed Fe-S cluster. In this way, neither the transferring protein nor the downstream target would retain the Fe-S cluster. Similarly, in spite of a high level of Fe-S cluster loss in Grx4, there was no activation of the Fe regulon. On the basis of a report where the DNA-binding activity of the Fe-responsive transcription factor Aft2 was explored *in vitro* (54), both Fe^2+^ metals and other divalent metals, such as Cd^2+^ metals, were able to disrupt the DNA binding activity of the transcription factor. Thus, it is possible that either Fe released from the Cu-disrupted Fe-S clusters or Cu itself could bind this transcription factor and its homolog Aft1 to prevent active transcription of their target genes.

**FIG 8:**
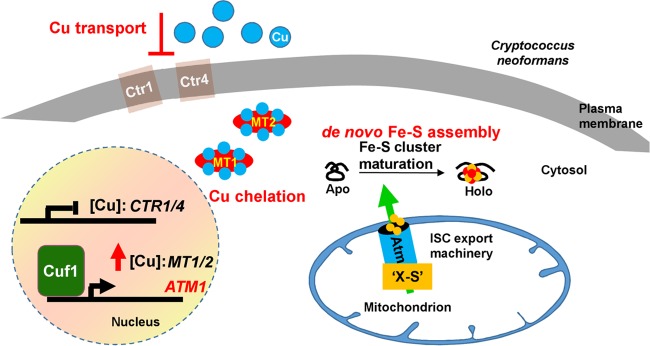
Model for the role of Atm1 during Cu stress in *C. neoformans*. In *C. neoformans*, Cuf1 is the primary transcription factor that regulates the expression of genes required for maintaining Cu homeostasis, either when Cu is scarce or when the concentrations approach toxic levels. When Cu concentrations approach toxic levels, Cuf1 occupancy is no longer enriched at promoters of genes with roles in Cu acquisition, as exemplified by the Cu importers Ctr1 and Ctr4, preventing the acquisition of more Cu^+^ from the environment. At the same time, Cuf1 occupancy is enriched at the *MT1* and *MT2* promoters, inducing the expression of the Mt1 and Mt2 proteins which sequester Cu, as they have high affinity and high binding capacity for this metal. The Cuf1-dependent and Cu-dependent induction of the expression of the mitochondrial ABC transporter Atm1 with a role in cytosolic and nuclear Fe-S protein biogenesis provides a novel way for coping with Cu toxicity. Fe-S proteins, which have many important and essential cellular functions, are major targets for Cu toxicity. Cu activation of Atm1 expression contributes to preservation of the homeostasis of cytosolic-nuclear Fe-S proteins, in particular, those with essential cellular functions, in the presence of toxic amounts of this metal.

*C. neoformans* is an environmental fungus and an opportunistic pathogen. For a successful life cycle, this organism requires a degree of plasticity for fast adaptation to different environments (7). In the case of Cu, environmental challenges might have favored the evolution of several layers of mechanisms for protecting *C. neoformans* from Cu stress, including an adaptation to both Cu deprivation and Cu toxicity (Fig. 8). When Cu levels approach toxic concentrations, *C. neoformans* prevents more Cu import into the cells by modifying both the localization and the stability of the Cu importers Ctr1 and Ctr4 at the plasma membrane, together with the release of Cuf1 from the Cu importer promoters (10, 17). Additionally, Cuf1 is enriched at the *MT1* and *MT2* promoters, inducing the expression of these cysteine-rich proteins, which can bind up to 16 and 24 atoms of Cu per protein, respectively (64). Despite the strong Cu-buffering capacity of the Mt proteins, this may be insufficient, and an additional Cuf1-dependent mechanism is elaborated to deal with Cu. The increased expression of the mitochondrial ABC transporter Atm1 serves as a novel mechanism to cope with Cu stress by increasing the export of an Fe-S cluster precursor(s) so that essential cellular processes which depend on the activities of Fe-S proteins remain active during Cu stress. Indeed, while Atm1 depletion modestly reduced the virulence of the fungus in an intranasal model of cryptococcosis, activated macrophages were more efficient in containing the growth of cells with low levels of Atm1 than control cells. These results likely reflect the importance of Atm1, and of an arsenal of essential Fe-S proteins, for the fitness of the pathogen in an *in vivo* setting.

## MATERIALS AND METHODS

### Strains and media.

*Cryptococcus neoformans* strains (see Table S1 in Text S1 in the supplemental material) were routinely grown at 30°C in synthetic complete (SC) medium (MP Biomedicals) containing either 2% glucose (SC or SC-gluc) or 2% galactose (SC-gal) or in yeast nitrogen base (YNB) medium containing either 2% glucose (YNB-gluc) or 2% galactose (YNB-gal), as indicated.

10.1128/mBio.01742-17.1 TEXT S1  Supplemental Materials and Methods. Download TEXT S1, PDF file, 0.3 MB. Copyright © 2017 Garcia-Santamarina et al. 2017 Garcia-Santamarina et al. This content is distributed under the terms of the Creative Commons Attribution 4.0 International license.

*S. cerevisiae* strains used in this study are listed in Table S1 in Text S1. Cells were cultivated in rich medium (YP) or SC supplemented with amino acids as required and 2% (wt/vol) glucose or galactose (65). Fe-depleted minimal medium was prepared using YNB without FeCl_3_ (Formedium). Plasmids used in this study are listed in Table S2 in Text S1. Plasmid constructs were verified by DNA sequencing and/or functional complementation of a corresponding yeast mutant.

### RNA isolation and qRT-PCR.

*C. neoformans ATM1* expression was analyzed in wild-type and *ΔCuf1* backgrounds as follows. Overnight cultures of *C. neoformans* in SC medium were inoculated into 5 ml fresh SC medium at an optical density at 600 nm (OD_600_) of 0.3 and allowed to grow for 2 h at 30°C with shaking at 200 rpm. Cultures were then treated with either 1 mM Cu_2_SO_4_ or 1 mM BCS during 3 h. After the treatment, cells were pelleted and stored at −80°C prior to processing. For gene expression in Atm1-F and Gal7-Atm1-F strains, cells were back-diluted in either SC-gluc medium or SC-gal medium (as indicated) for three consecutive days. At day 3, cells were diluted (in each corresponding medium) to an OD_600_ of 0.3 and were allowed to grow for 2 h as described above. After the 2 h had elapsed, cells were either left untreated or treated with the indicated amounts of CuSO_4_ for 3 h. Cells were then pelleted and stored at −80°C prior to processing. RNA was isolated from the stored pellets using a Qiagen RNeasy minikit, and DNase was treated with a Turbo DNA-free kit (Roche). Quantitative PCR (qPCR) was performed using specific primers for *ACT1*, *ATM1*, *CIG1*, *SIT1*, *MT1*, and *MT2* (primer sequences are described in Table S3 in Text S1) and iQ SYBR green Supermix (BioRad), and amplification and detection were performed using a CFX384 real-time system (BioRad), as previously described (66). Normalized expression levels were determined by calculating the threshold cycle (ΔΔ*C*_*T*_) value for each gene in relation to *ACT1*.

### Chromatin immunoprecipitation.

Cells expressing Cuf1-FLAG were treated with 1 mM Cu or 1 mM BCS for 3 h. ChIP-PCR analysis was performed as described previously (9). Promoter sequences of *ATM1* and *TUB2* were analyzed by qPCR. The primers used are described in Table S3 in Text S1.

### Protein extraction and Western blotting.

For analyzing Atm1-FLAG expression, overnight SC cultures of *C. neoformans* were diluted to an OD_600_ of 0.3 in 5 ml of fresh SC media. Cells were grown for 2 h more and then treated with the indicated concentrations of Cu_2_SO_4_ for the indicated times. After each time point, 100% trichloroacetic acid (TCA) was added to the culture to reach a 10% concentration and cells were pelleted and collected with 20% TCA and stored at −20°C. To check the sugar-dependent expression of Atm1 and Leu1-HA expression after ZPT and/or Cu treatments in the Atm1-F and Gal7-Atm1-F strains, cells were back-diluted in either SC-gal or SC-gluc medium (as indicated) for three consecutive days (as indicated). At day 3, cells were diluted in 5 ml of fresh SC-gal or SC-gluc medium to an OD_600_ of 0.3. After 3 additional hours of growth, 100% TCA was added to reach a 10% final concentration and cells were collected and stored as described above. Stored pelleted cells were treated with 100 μl of 10% TCA, approximately 100 μl of glass beads was added, and the pellets were disrupted in a Mini-BeadBeater (BioSpec) using three sets of 1-min-ON and 1-min-OFF intervals. The whole-cell extract (without the beads) was transferred to a new tube, and the reaction mixture was centrifuged at maximum speed for 2 min in a microcentrifuge. The supernatant was discarded, and the cellular pellet was washed with 1 ml of ice-cold acetone. After 2 min of centrifugation, the acetone was eliminated and the pellet was dried and then resuspended in 80 μl of 100 mM Tris-HCl (pH 8.3)–1 mM EDTA–1% SDS. Samples were loaded in a gel and were analyzed with FLAG (monoclonal and horseradish peroxidase [HRP] conjugated; Sigma-Aldrich), H3 (D1H2, polyclonal; Cell Signaling Technology, Inc.), HA (Y-11, polyclonal; Santa Cruz), Aco, or Cdc2 (monoclonal, anti-PSTAIR; Abcam, Inc.) antibodies.

### Enzymatic activities.

For measurement of enzymatic activities, cells were back-diluted in SC-gluc medium for three consecutive days. At day 3, cells were diluted to an OD_600_ of 0.3 in fresh SC-gluc media and were allowed to grow for 2 h. At that time point, cells were either left untreated or treated at the indicated concentrations of stressors for the indicated times, and the enzymatic activities were measured as previously described (67).

### Microscopy.

*C. neoformans* cells expressing or not expressing Atm1-mCherry from the *GAL7* promoter were back-diluted in SC-gal medium for three consecutive days. At day 3, cells were diluted to an OD_600_ of 0.3 in fresh SC-gal media and allowed to grow for 2 h. At that time point, cells were stained with Mitotracker green (Invitrogen) following the instructions of the manufacturer. Microscopy pictures were taken using a Zeiss 780 upright fixed-stage confocal microscope.

### Growth curves.

Quantitative growth analysis was performed to analyze the susceptibility of strains to stressors as follows. *C. neoformans* cultures were back-diluted during three consecutive days either in galactose (SC-gal or YNB-gal) or in glucose (SC-gluc or YNB-gluc) medium. At day 3, cells were diluted to an OD_600_ of 0.002, in fresh media, and were divided into aliquots and added to 96-well plates. Test compounds were added from fresh stock solutions of Cu_2_SO_4_ prepared in water and of 1-hydroxypyridine-2-thione zinc salt (zinc-pyrithione or ZPT) prepared in dimethyl sulfoxide (DMSO) (all products from Sigma-Aldrich), at the indicated concentrations. Plates were covered with a semipermeable membrane and incubated at 30°C with shaking at 900 rpm in a Finstruments shaker instrument. Growth graphs were generated by plotting the OD_600_ readings versus the compound concentrations at the 48-h time point.

### Miscellaneous methods.

The following published methods were used: manipulation of DNA and PCR (68); transformation of *S. cerevisiae* cells (69); determination of promoter strength using luciferase (67); and *in vivo* labeling of *C. neoformans* or *S. cerevisiae* cells with ^55^FeCl (ICN) and measurement of ^55^Fe incorporation into Fe-S proteins by immunoprecipitation and scintillation counting (67, 70). For *S. cerevisiae* Western blot analysis, antibodies were raised against recombinant purified proteins in rabbits. Antibodies against c-Myc or HA were obtained from Santa Cruz and protein A Sepharose from GE Healthcare.

### Macrophage coculturing assay.

The macrophage coculturing assay was performed as previously described (66). After 2 h of coinfection, nonphagocytosed *C. neoformans* cells were removed, and the remaining macrophages (containing or not containing *C. neoformans*) were washed 3 times with phosphate-buffered saline (PBS). Half of the coinfection cultures were plated for *C. neoformans* CFU at this point (the 2-h time point for calculating the phagocytosis index), and the other half were kept for an additional 24 h before plating for CFU (24-h time point for calculating the percentage of *C. neoformans* survival) was performed. *C. neoformans* cells for CFU were plated in SC-gal plates and incubated for up to 72 h at 30°C.

### Mouse infection experiment.

Female A/J mice (aged 4 to 6 weeks) were purchased from Jackson Laboratories. Mice were given a week to acclimate at the mouse facility. For the animal infection, the Atm1-F and the Gal7-Atm1-F strains were grown in SC-gluc medium for two consecutive days. At day 2, cells were washed with PBS three times and diluted to a cellular density of 4 × 10^6^ cell/ml. Twenty-five microliters of this dilution was intranasally inoculated into mice that had previously been anesthetized in a manner consistent with IACUC standards. The mouse endpoint was considered a 15% reduction in weight.

### Statistical analysis.

In all figures, error bars represent statistical errors of the means (SEM) of results from a number of biological replicates (*n*), as indicated in the figure legends. The SEM was used because it provides a measurement of the accuracy of means of results of comparisons between different biological replicates. Before any statistical tests were conducted, data were log transformed for comparisons of proportions. The statistical tests chosen were paired *t* tests or analysis of variance (ANOVA) for repeated measures. The rationale for using paired and repeated-measures tests is that the experimental samples within a biological replicate are paired by experimental day. Data corresponding to results of statistical tests and the calculated *P* values are indicated in the figure legends. For those *P* values calculated with ANOVA which were significant, either the Bonferroni test or Fisher’s least-significant-difference test was applied to find differences within the groups (multiple-comparison tests) and the data were labeled with asterisks corresponding to statistical significance as follows: ****, *P* = <0.0001; ***, *P* = 0.0001 to *P* = <0.001; **, *P* = 0.001 to *P* = <0.01; *, *P* = 0.01 to *P* = <0.05; ns (not significant), *P* = >0.05. Nonsignificant values are either not indicated or indicated for relevant experiments only.
