# Supplementary figures and images for: Cryptococcus neoformans Iron-Sulfur Protein Biogenesis Machinery Is a Novel Layer of Protection against Cu Stress
Source: mBio. 2017 Oct 31;8(5):e01742-17. doi: 10.1128/mBio.01742-17 (PMC5666163; doi:10.1128/mBio.01742-17)

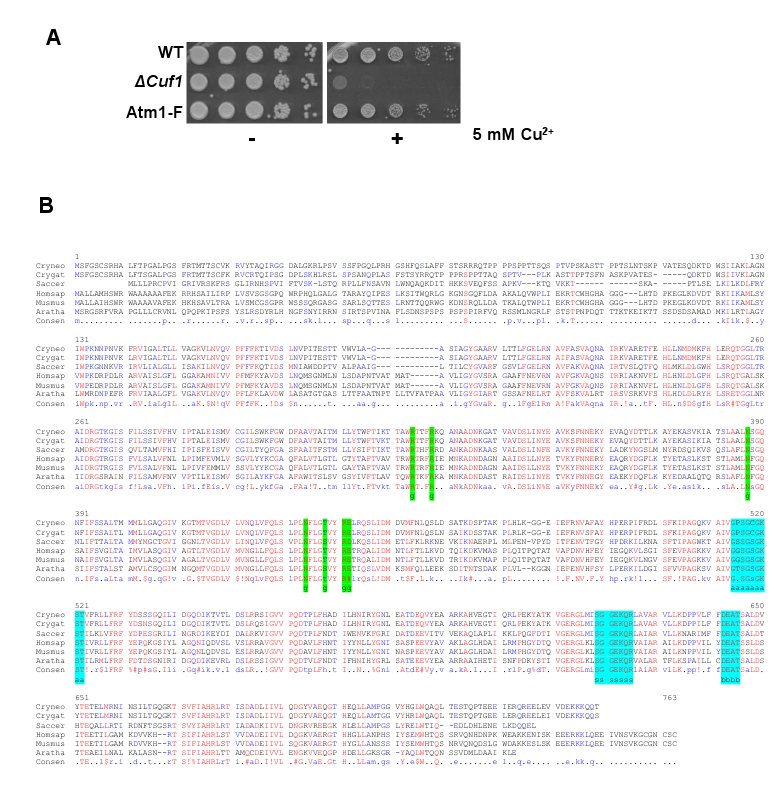

Supplement: FIG S1 [file mbo005173572sf1.tif]

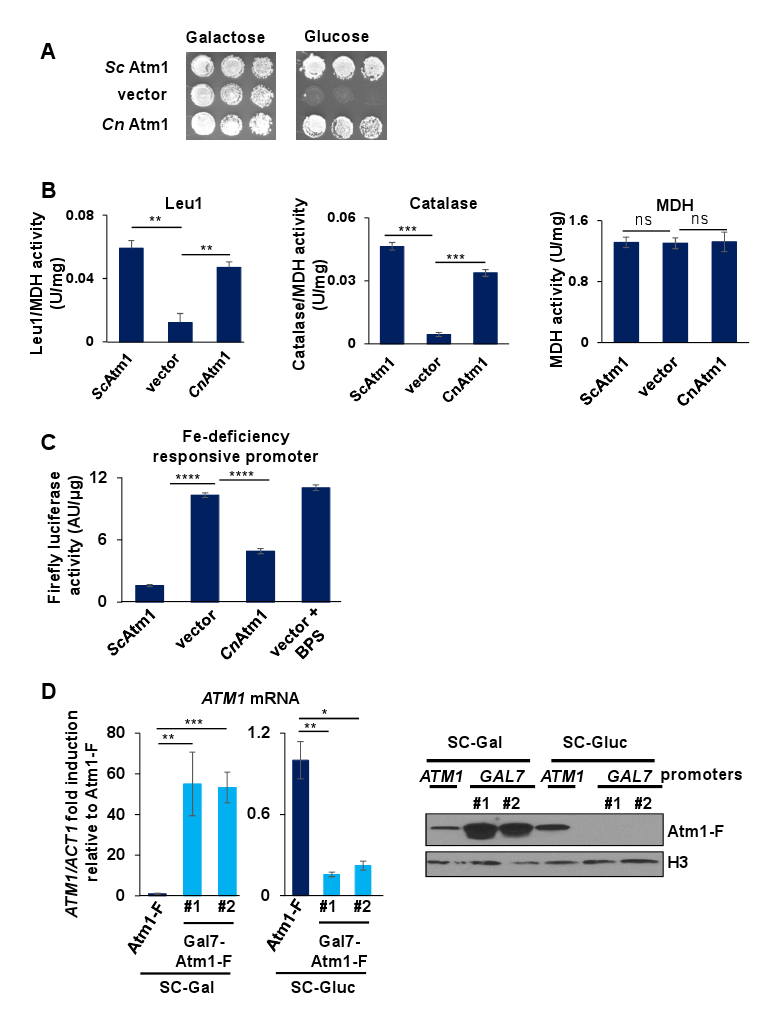

Supplement: FIG S2 [file mbo005173572sf2.tif]

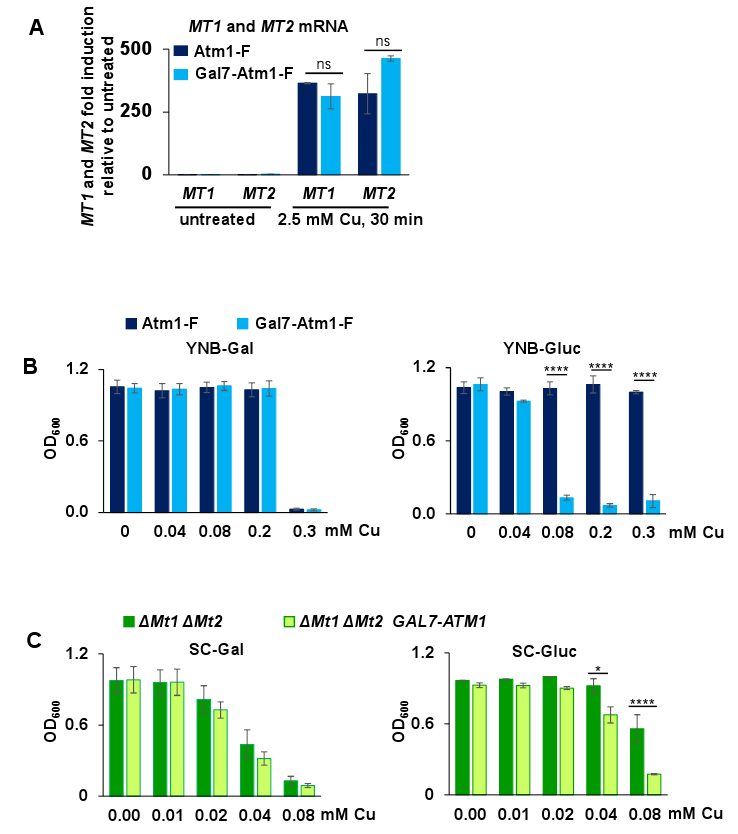

Supplement: FIG S3 [file mbo005173572sf3.tif]

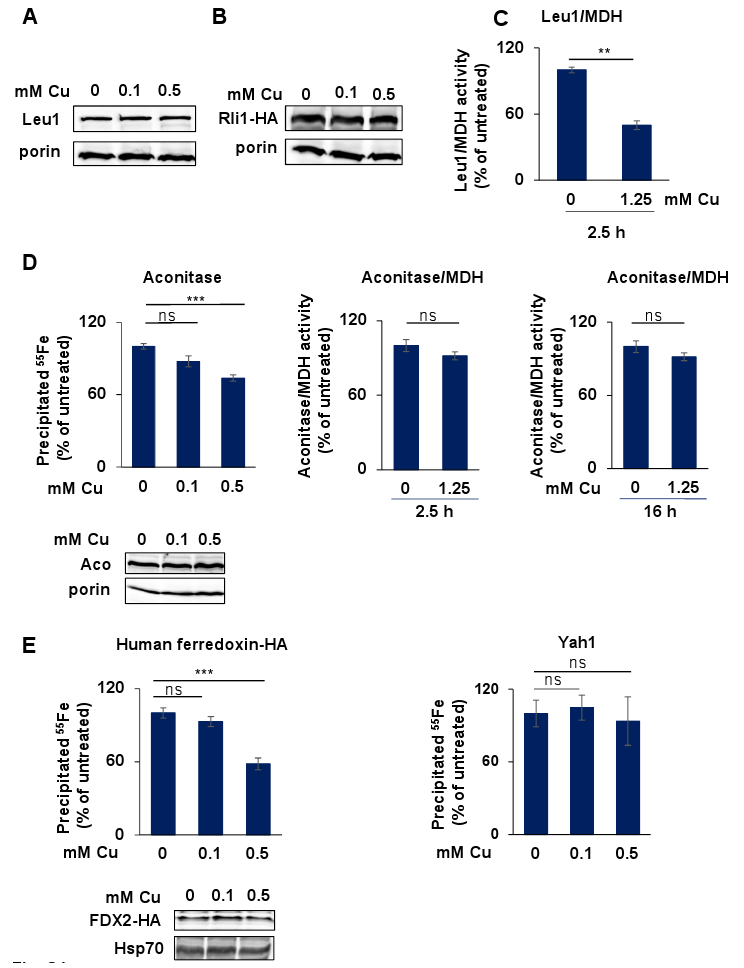

Supplement: FIG S4 [file mbo005173572sf4.tif]

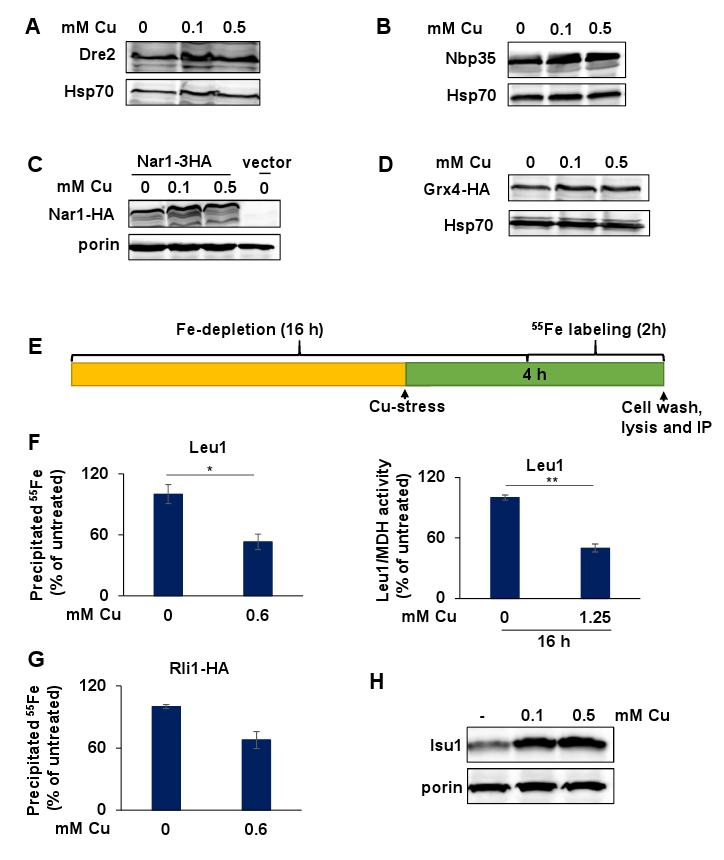

Supplement: FIG S5 [file mbo005173572sf5.tif]

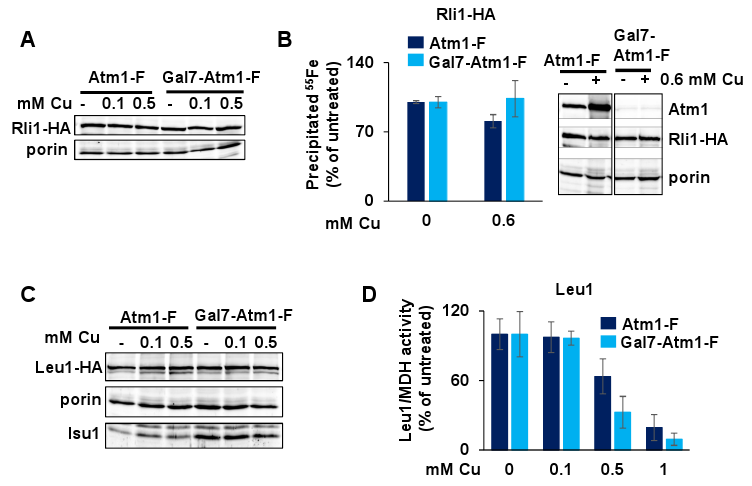

Supplement: FIG S6 [file mbo005173572sf6.tif]

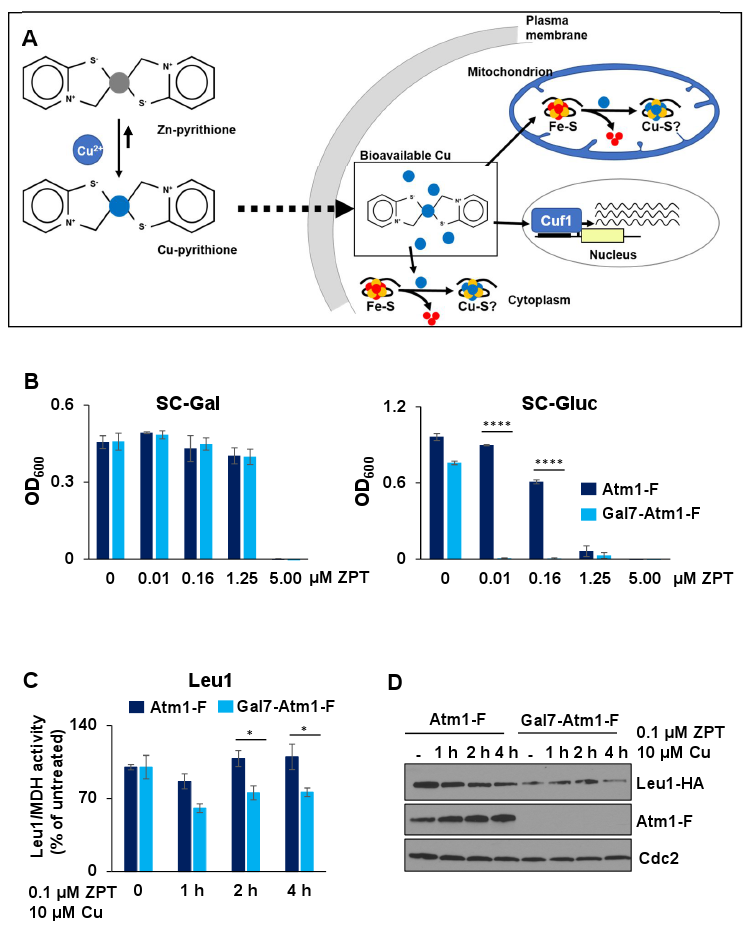

Supplement: FIG S7 [file mbo005173572sf7.tif]

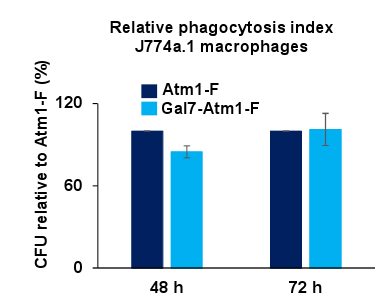

Supplement: FIG S8 [file mbo005173572sf8.tif]
