# Supplementary material for: Cryptococcus neoformans Iron-Sulfur Protein Biogenesis Machinery Is a Novel Layer of Protection against Cu Stress
Source: mBio. 2017 Oct 31;8(5):e01742-17. doi: 10.1128/mBio.01742-17 (PMC5666163; doi:10.1128/mBio.01742-17)
Supplement: TEXT S1 [file mbo005173572s1.pdf]

SUPPLEMENTAL MATERIALS AND METHODS

Includes Supplemental Materials and Methods and Table S1. List of strains used in this study, Table S2. List of plasmids used in this study, and Table S3. List of plasmids used in this study.

Generation of *Cryptococcus neoformans* mutants

YPD, supplemented with 1.5% agar and 100 µg ml-1 of nourseothricin (NAT), 200 µg ml-1 of neomycin (G418) or 200 u ml-1 of hygromycin B (HYG) was used for colony selection after biolistic transformation.

The *C. neoformans* Atm1-F strains (wimp and stud) (DTY947 and DTY967, respectively) were generated as follows: the multiple cloning site of pSL1180 (Amersham) was amplified with the primer pair OLSG-49 and OLSG-50 (primers sequences are described in Table S2), and HindIII/EcoRI cloned into pUC19 to yield plasmid pDT1799. The *NAT* cassette from pAI3 (1) was amplified with the primer pair OLSG-35 and OLSG-36 and NheI cloned into DTP1799 to generate the plasmid pDT1800. The *4FLAG-HOG1* terminator sequence was amplified from plasmid p553 (gift from Dr. Sun-Bahn, Yonsei University, Seoul) with the primer pair OLSG-158 and OLSG-107 and cloned into pDT1800 with the restriction sites SacII/StuI to generate the plasmid pDT1802. The primer pair OLSG-196 and OLSG-197 was used to amplify

approximately 1kb upstream of the stop codon (not including the stop codon) of *ATM1* (ATM1up) using genomic DNA as a template. This fragment was digested with AscI and cloned into pDT1801 to generate the plasmid pDT1802. The primer pair OLSG-93 and OLSG-94 was used to amplify approximately 1kb downstream of the stop codon (not including the stop codon) of *ATM1* (ATM1d) using genomic DNA as a template. This fragment was digested with XmaI and cloned into pDT1802 to generate the plasmid pDT1803. A PCR product, including the sequence ATM1up-4FLAG-HOGt-NAT-ATM1d was obtained with the primer pairs OLSG-276 and OLSG-54, using the plasmid pDT1803 as a template, and transformed in *C. neoformans* strains H99 wimp (DTY758) and H99 stud (DTY981) by biolistic transformation, to generate the strains DTY947 and DTY967, as previously described.

The *C. neoformans* strains Gal7-Atm1-F wimp and stud (DTY949 and DTY969, respectively) strains were generated as follows: the G418 cassette from pJAF1 (2) was amplified with the primer pair OLSG-112 and OLSG-113 and NheI cloned into pDT1799 to generate the plasmid pDT1804. A fragment of approximately 1kb was amplified from the genomic DNA using OLSG-294 and OLSG-295 as a primer pair. This fragment was cut with the restriction enzymes SacII and StuI, and cloned into pDT1804 to generate the plasmid pDT1805. A PCR product amplifying the *GAL7* promoter was obtained with OLSG-297 and OLSG-298, using pCN69 as a template, a second PCR product, with size approximately 1kb, and starting at the *ATM1* initiation codon, was obtained with the primer pair OLSG-298 and OLSG-299 using genomic DNA as a template. These two PCR products were cloned into pDT1805 using the infusion cloning kit (Clontech

Laboratories) to generate the plasmid pDT1806. The plasmid was linearized with SacII, and transformed in both *C. neoformans* DTY947 to generate the strain DTY949, and in *C. neoformans* DTY967 to generate the strain DTY969, by biolistic transformation.

The *C. neoformans* strain Gal7-Atm1-mCherry (DTY951) strain was generated according to the following steps: the mCherry-HOGt sequence was amplified from p524 (gift from Dr. Sun-Bahn) with OLSG-194 and OLSG-195, and cloned after digestion with the restriction enzymes SacII/NgoMIV into pDT1800. The primer pair OLSG-196 and OLSG-197 was used to amplify approximately 1kb upstream of the stop codon (not including the stop codon) of *ATM1* (ATM1up) using genomic DNA as a template. This fragment was digested with AscI and cloned into pDT1807 to generate the plasmid pDT1808. The primer pair OLSG-93 and OLSG-94 was used to amplify approximately 1kb downstream of the stop codon (not including the stop codon) of *ATM1* (ATM1d) using genomic DNA as a template. This fragment was digested with XmaI and cloned into pDT1808 to generate the plasmid pDT1809. A PCR product, including the sequence ATM1up-mCherry-HOGt-NAT-ATM1d was obtained with the primer pairs OLSG-276 and OLSG-277 and transformed in *C. neoformans* strain H99 (DTY758) by biolistic transformation to generate the strain ATM1-mCherry. A maxiprep for pDT1806 was obtained, the plasmid was linearized with SacII, and transformed in Atm1-mCherry by biolistic transformation, to generate the Gal7-Atm1-mCherry strain.

C. neoformans Atm1-F Rli1-HA (DTY955) and Gal7-Atm1-F Rli1-HA (DTY957) strains were generated as follows: the primer pair OLSG-190 and OLSG-191 was used to amplify the *HYG* cassette from the pHYG-KB1 vector (3), the PCR fragment obtained was digested with KpnI and cloned into pDT1799 to generate the pDT1810 vector. The Rli1-HA-Hog terminator sequence, including a SbfI site between the RLI1 sequence and the HA, was synthesized by Integrated DNA Technologies (IDT), this sequence was amplified with OLSG-358 and OLSG-107 and digested with the restriction enzymes XhoI/StuI for cloning it into pDT1810 vector, to generate the pDT1811 vector. A PCR fragment, approximately 1kb downstream of the stop codon (and not including the stop codon) was amplified with OLSG-359 and OLSG-360 and cloned into pDT1811 after digestion with XmaI/XbaI to generate the vector pDT1812. A maxiprep of pDT1812 was obtained, and the harvested plasmid was digested with XhoI for linearization before biolistic transformation in both DTY947 to obtain the strain Atm1-F Rli1-HA (DTY955) and in DTY949 to obtain the strain Gal7-Atm1-F Rli1-HA (DTY957).

C. neoformans Atm1-F Leu1-HA (DTY959) and Gal7-Atm1-F Leu1-HA (DTY961) strains were generated as follows: the primer pair OLSG-412 and OLSG-413 was used to amplify approximately 1kb upstream the *LEU1* stop codon (without including it), the PCR product obtained was digested with SpeI/SbfI and cloned into pDT1811 SpeI/SbfI digested to yield the vector pDT1813. A PCR fragment, approximately 1kb downstream of the stop codon (and not including the stop codon) was amplified with OLSG-414 and OLSG-415 and cloned into DTP1813 after digestion with XbaI/BamHI to generate the vector pDT1814. A PCR product was obtained by amplifying the sequence LEU1up-HA-

HOGt-HYG-LEU1d with OLSG-434 and OLSG-54 and transformed in both DTY947 to obtain the strain Atm1-F Leu1-HA (DTY959) and in DTY949 to obtain the strain Gal7-Atm1-F Leu1-HA (DTY961).

C. neoformans Atm1-F Lys4-HA (DTY963) and Gal7-Atm1-F Lys4-HA (DTY965) strains were generated as follows: the primer pair OLSG-445 and OLSG-446 was used to amplify approximately 1kb upstream the *LYS4* stop codon (without including it), the PCR product obtained was cloned with the infusion kit (Clontech Laboratories) into DTP1811 SpeI/SbfI digested to yield the vector pDT1815. A PCR fragment, approximately 1kb downstream of the stop codon (and not including the stop codon) was amplified with OLSG-447 and OLSG-448 and cloned into pDT1815 after digestion with XbaI/BamHI to generate the vector pDT1816. A PCR product was obtained by amplifying the sequence LYS4up-HA-HOGt-HYG-LYS4d with OLSG-465 and OLSG-54 and transformed in both DTY947 to obtain the strain Atm1-F Lys4-HA (DTY963) and in DTY949 to obtain the strain Gal7-Atm1-F Lys4-HA (DTY965). Primers are described in Table S3.

REFERENCES TO THE SUPPLEMENTAL MATERIALS AND METHODS

1. Idnurm A, Reedy JL, Nussbaum JC, Heitman J. 2004. Cryptococcus neoformans virulence gene discovery through insertional mutagenesis. Eukaryot Cell 3:420-9.
2. Fraser JA, Subaran RL, Nichols CB, Heitman J. 2003. Recapitulation of the sexual cycle of the primary fungal pathogen Cryptococcus neoformans var. gattii: implications for an outbreak on Vancouver Island, Canada. Eukaryot Cell 2:1036-45.
3. Hua J, Meyer JD, Lodge JK. 2000. Development of positive selectable markers for the fungal pathogen Cryptococcus neoformans. Clin Diagn Lab Immunol 7:125-8.

Table S1. List of strains used in this study

NAME	YEAST	STRAIN	GENOTYPE	REFERENCE
DTY758	*C. neoformans*	H99	wild-type wimp	J. Heitman lab, Duke University, USA
DTY981	*C. neoformans*	H99	wild-type stud	J. Heitman lab, Duke University, USA
DTY761	*C. neoformans*	*ΔCuf1*	*cuf1::Neo* wimp	(1)
DTY762	*C. neoformans*	*ΔCuf1* + pCuf1-FLAG	*cuf1::Neo/CUF1-FLAG::HYG* wimp	(2)
DTY947	*C. neoformans*	Atm1-F	*ATM1-4FLAG::NAT* wimp	This study, derived from DTY758
DTY949	*C. neoformans*	Gal7-Atm1-F	*ATM1p::GAL7p::G418 ATM1-4FLAG::NAT* wimp isolate #1	This study, derived from DTY947
DTY949	*C. neoformans*	Gal7-Atm1-F	*ATM1p::GAL7p::G418 ATM1-4FLAG::NAT* wimp isolate #2	This study, derived from DTY947
DTY951	*C. neoformans*	Gal7-Atm1-mCherry	*ATM1p::GAL7p::G418 ATM1-mCherry::NAT* wimp	This study
DTY756	*C. neoformans*	*ΔMt1 ΔMt2*	*mt1::NAT mt2::NEO* wimp	(1)
DTY953	*C. neoformans*	*ΔMt1 ΔMt2* Gal7-Atm1	*mt1::NAT mt2::NEO ATM1p::GAL7p::G418* wimp	This study, derived from DTY756
DTY955	*C. neoformans*	Atm1-F Rli1-HA	*ATM1-4FLAG::NAT RLI1-HA::HYG* wimp	This study, derived from DTY947
DTY957	*C. neoformans*	Gal7-Atm1-F Rli1-HA	*ATM1p::GAL7p::G418 ATM1-4FLAG::NAT RLI1-HA::HYG* wimp	This study, derived from DTY949
DTY959	*C. neoformans*	Atm1-F Leu1-HA	*ATM1-4FLAG::NAT LEU1-HA::HYG* wimp	This study, derived from DTY947
DTY961	*C. neoformans*	Gal7-Atm1-F Leu1-HA	*ATM1p::GAL7p::G418 ATM1-4FLAG::NAT LEU1-HA::HYG* wimp	This study, derived from DTY949
DTY963	*C. neoformans*	Atm1-F Lys4-HA	*ATM1-4FLAG::NAT LYS4-HA::HYG* wimp	This study, derived from DTY947
DTY965	*C. neoformans*	Gal7-Atm1-F Lys4-HA	*ATM1p::GAL7p::G418 ATM1-4FLAG::NAT LYS4-HA::HYG* wimp	This study, derived from DTY949
DTY967	*C. neoformans*	Atm1-F	*ATM1-4FLAG::NAT* stud	This study, derived from DTY946
DTY969	*C. neoformans*	Gal7-Atm1-F	*ATM1p::GAL7p::G418 ATM1-4FLAG::NAT* stud	This study, derived from DTY946
	S. cerivisiae	GalL-Atm1	*MATa, ura3-1, ade2-1, trp1-1, his3-11,15 leu2-3,112 ATM1p::GalLp*	(3)
	S. cerivisiae	GalL-Atm1 Aft1-lux	*MATa, ura3-1, ade2-1, trp1-1, his3-11,15 leu2-3,112 ATM1p::GalLp AFT1::HIS3 AFT1lux*	Dr U. Muehlenhoff, Philipps-Universitat Marburg
	S. cerivisiae	Grx4-HA	*MATa, ura3-1, ade2-1, trp1-1, his3-11,15 leu2-3,112::GRX4-HA tetO2* promoter	(4)

REFERENCES

(1) Ding C. et al. (2011) The Copper regulon of the human fungal pathogen Cryptococcus neoformans H99. Molecular Microbiology 81(6), 1560-1576

(2) Ding C. et al. (2013) *Cryptococcus neoformans* copper detoxification machinery is critical for fungal virulence. Cell Host and Microbe 13, 265-276

(3) Srinivasan V, et al. (2014) Crystal structures of nucleotide-free and glutathione-bound mitochondrial ABC transporter Atm1. Science 343(6175):1137-40

(4) Molina MM et al. (2004) Nuclear monothiol glutaredoxins of Saccharomyces cerevisiae can function as mitochondrial glutaredoxins. J Biol Chem 279(50):51923-30

Table S2. List of plasmids used in this study

PLASMID	ORF	BACKBONE	REFERENCE
pRS416-NP-ScAtm1	ScATM1, MET25 promoter replaced by ATM1p	pRS416-MET25	(1)
p416-pGPD-CnAtm1	*C. neoformans* ATM1 cDNA under control of GPD promoter	p416-GPD	This work
p416-pCCC1-LUX	luc2 under control of CCC1 (-600 to -1)	p416-Luc2	(2)
p426-FDX2-HA	FDX2 (Homo sapiens); C-terminal HA	p426-TDH3	(3)
p426-NAR1-HA	NAR1, C-terminal HA	p426-TDH3	(4)
p424-NBP35-TAP	NBP35, C-terminal TAP	p424-TDH3	(5)
p426-RLI1-HA	RLI1; C-terminal HA; own promoter replacing TDH3 promoter	p426-TDH3	(6)
p414-SDH2-Myc	SDH2; C-terminal Myc	p414-MET25	D.R. Winge, University of Utah, USA
p416-SDH1	SDH1	p416-MET25	D.R.Winge, University of Utah, USA

REFERENCES

(1) Srinivasan V, et al. (2014) Crystal structures of nucleotide-free and glutathione-bound mitochondrial ABC transporter Atm1. Science, 343(6175):1137-40

(2) Rietzschel N, et al. (2015) The Basic Leucine Zipper Stress Response Regulator Yap5 Senses High-Iron Conditions by Coordination of [2Fe-2S] Clusters. Mol Cell Biol 35(2):370-8

(3) Sheftel AD, et al. (2010) Humans possess two mitochondrial ferredoxins, Fdx1 and Fdx2, with distinct roles in steroidogenesis, heme, and Fe/S cluster biosynthesis. PNAS 107 (26) 11775-11780

(4) Hausman A, et al. (2005) The eukaryotic P loop NTPase Nbp35: an essential component of the cytosolic and nuclear iron-sulfur protein assembly machinery. PNAS 102(9): 3266-3271

(5) Netz DJ, et al. (2012) A bridging [4Fe-4S] cluster and nucleotide binding are essential for function of the Cfd1-Nbp35 complex as a scaffold in Fe-S protein maturation. J Biol Chem (287):15 [illegible].

(6) Kispal G, et al. (2005) Biogenesis of cytosolic ribosomes requires the essential iron-sulphur protein Rli1p and mitochondria. EMBO J 24(3):589-98

Table S3. List of primers used in this study

PRIMER NAME	SEQUENCE	COMMENTS
OLSG-35	ACGGCTAGCGTAAAACGACGGCCAGT	
OLSG-36	ACGGCTAGCAACAGCTATGACCATG	
OLSG-49	ACGAAGCTTAAGGTGCACGGCCC	
OLSG-50	CATGAATTCGAATGGCCATGGGAC	
OLSG-54	CAGCTGGCGAAAGGGGGATGT	
OLSG-93	ATGCCCGGGACTTAGAAGAAGAGGTTGG	
OLSG-94	ATGCCCGGGAATTATAGTTCACTTTCTC	
OLSG-107	ATGAGGCCTCAATCTATCCCTCTCTCC	
OLSG-112	ACGGCTAGCGACTCACTATAGGGCG	
OLSG-113	ACGGCTAGCCCACTAGTAACGGCCGC	
OLSG-158	ATGCCGCGGGGCGCGCCAGATTACAAGGATGACGAC	
OLSG-190	ATGGGTACCACTCGCTATTGTCCAGGCTG	
OLSG-191	ATGGGTACCGCAACCATGTGATGTGCAAA	
OLSG-194	TAGCCGCGGGGCGCGCCAGGTGGCGGTGGCTCTGTGA	
OLSG-195	ATGGCCGGCCAATCTATCCCTCTCTCCGA	
OLSG-196	ACGGGCGCGCCTTCCCTTCCCCTCAACTTCC	
OLSG-197	ACGGGCGCGCCCTGCTTTTTCTTCTCGTCCA	
OLSG-276	CAGTCTACCGCGAACTTCGTCAAA	
OLSG-277	TTGGGAAGGGCGATCGGTG	
OLSG-294	ATGCCGCGGACTCACTATGATCGAAAAAG	
OLSG-295	ATCAGGCCTAATGGTCGCTGACATTCTAC	
OLSG-296	CATTGCAATTGGATCCGGTACCGAGCTCGAGCTC	
OLSG-297	GGAGCCGAAGCCCATTCTCAGGAGAGAATTGAGTG	
OLSG-298	ATGGGCTTCGGCTCCTGC	
OLSG-299	AGGTAATTATAACCCGGGATGGTAATCGCAGTAACGG	
OLSG-358	ATTCTCGAGCATGACCAAGACTCTTGGCAAC	
OLSG-359	ATTTCTAGAAGCGATGAATCAAAAACCTGC	
OLSG-360	TTACCCGGGGATCAGATTCGTTTTCAGC	
OLSG-412	ACGACTAGTAACTTCGAAGGTCGACAAGGC	
OLSG-413	ATCCCTGCAGGCCAGTCCGTCTTCCTGACGCC	
OLSG-414	ACTTCTAGAGGGCAGATTGGAGATAATGGC	
OLSG-415	ACTGGATCCGCCGTAATGAGGCTTTCCGG	
OLSG-434	GTCGACAAGGCGCTGGTGGCC	
OLSG-445	CGGCCCACGTGGCCACTAGTACTTTGATTGACGCA	
OLSG-446	ACCGGCGGCCATTCCTGCAGGTAATGAAGCTCTAA	
OLSG-447	TCCTCTAGAGCATTTGTCATGATTAGAGC	
OLSG-448	TATGGATCCTTCATCACCAGGATCTCCAC	
OLSG-465	TGCAGGCTGCGGTCCTTGTATTG	
OLSG-542	TGAGTGAAAGTGGCTCATCG	*TUB2* ChIP PRIMER FORWARD
OLSG-543	AGCAAGCCAAAAACAACACC	*TUB2* ChIP PRIMER REVERSE
OLRF-304	CGGGATTAACGTGATTGCTT	*ATM1* ChIP PRIMER FORWARD
OLRF-310	TAGAACGAGGAACGCGCTAT	*ATM1* ChIP PRIMER REVERSE
OLRF-272	GCACCATACCTTCTACAATGAGC	*ACT1* qRT-PCR PRIMER FORWARD
OLRF-273	CAGGAAGCTCGTAAGACTTTTCA	*ACT1* qRT-PCR PRIMER REVERSE
OLRF-260	TCATCTGGAGCGTCAAACTG	*ATM1* qRT-PCR PRIMER FORWARD
OLRF-261	GCCAAATTTCCATGAGAGGA	*ATM1* qRT-PCR PRIMER REVERSE
OLRF-350	TACGAGCGAGGCTCAAGACA	*MT1* qRT-PCR PRIMER FORWARD
OLRF-351	GGCAGCGCCAGATCCGCTG	*MT1* qRT-PCR PRIMER REVERSE
OLRF-352	TTCAACCCCAACCCAGAAAAG	*MT2* qRT-PCR PRIMER FORWARD
ORF-353	CACCTTGGCAACCGCATGTG	*MT2* qRT-PCR PRIMER REVERSE
OLSG-361	CACATTCACCGCTGCCATGGCTG	*CIG1* qRT-PCR PRIMER FORWARD
OLSG-362	CTGTTGACCGGGTCGAAACCAC	*CIG1* qRT-PCR PRIMER REVERSE
OLSG-363	ATGCCAGACCAGCTCTATCCG	*SIT1* qRT-PCR PRIMER FORWARD
OLSG-364	GACAGAGCCATCCAGGCCATAGC	*SIT1* qRT-PCR PRIMER REVERSE
